# Upstream Transcription Factor 1 (*USF1*) allelic variants regulate lipoprotein metabolism in women and USF1 expression in atherosclerotic plaque

**DOI:** 10.1038/srep04650

**Published:** 2014-04-11

**Authors:** Yue-Mei Fan, Jussi Hernesniemi, Niku Oksala, Mari Levula, Emma Raitoharju, Auni Collings, Nina Hutri-Kähönen, Markus Juonala, Jukka Marniemi, Leo-Pekka Lyytikäinen, Ilkka Seppälä, Ari Mennander, Matti Tarkka, Antti J. Kangas, Pasi Soininen, Juha Pekka Salenius, Norman Klopp, Thomas Illig, Tomi Laitinen, Mika Ala-Korpela, Reijo Laaksonen, Jorma Viikari, Mika Kähönen, Olli T. Raitakari, Terho Lehtimäki

**Affiliations:** 1 Department of Clinical Chemistry, Fimlab Laboratories, Tampere University Hospital and School of Medicine at the University of Tampere, Tampere; 2 Department of Pediatrics, University of Tampere and Tampere University Hospital, Tampere; 3 Research Centre of Applied and Preventive Cardiovascular Medicine, University of Turku, Turku; 4 Department of Health and Functional Capacity, Population Research Laboratory, National Public Health Institute; 5 Heart Center, Department of Cardiac Surgery, Tampere University Hospital, Finland; 6 Computational Medicine, Institute of Health Sciences, University of Oulu, Oulu, Finland; 7 NMR Metabolomics Laboratory, School of Pharmacy, University of Eastern Finland, Kuopio, Finland; 8 Oulu University Hospital, Oulu, Finland; 9 Divison of Vascular Surgery, Department of Surgery, Tampere University Hospital and University of Tampere, Finland; 10 Institute of Epidemiology, Helmholtz Center Munich, Munich, 85764, Germany; 11 Department of Clinical Physiology and Nuclear Medicine, Kuopio University Hospital and University of Eastern Finland, Finland; 12 Computational Medicine, School of Social and Community Medicine & Medical Research Council Integrative Epidemiology Unit, University of Bristol, Bristol, United Kingdom; 13 Department of Medicine, University of Turku and Division of Medicine, Turku University Hospital, Turku, Finland; 14 Department of Clinical Physiology, University of Tampere and Tampere University Hospital, Tampere; 15 Research Centre of Applied and Preventive Cardiovascular Medicine, University of Turku and Department of Clinical Physiology, University of Turku and Turku University Hospital, Turku, Finland

## Abstract

Upstream transcription factor 1 (*USF1*) allelic variants significantly influence future risk of cardiovascular disease and overall mortality in females. We investigated sex-specific effects of *USF1* gene allelic variants on serum indices of lipoprotein metabolism, early markers of asymptomatic atherosclerosis and their changes during six years of follow-up. In addition, we investigated the *cis*-regulatory role of these *USF1* variants in artery wall tissues in Caucasians. In the Cardiovascular Risk in Young Finns Study, 1,608 participants (56% women, aged 31.9 ± 4.9) with lipids and cIMT data were included. For functional study, whole genome mRNA expression profiling was performed in 91 histologically classified atherosclerotic samples. In females, serum total, LDL cholesterol and apoB levels increased gradually according to *USF1* rs2516839 genotypes TT < CT < CC and rs1556259 AA < AG < GG as well as according to *USF1* H3 (GCCCGG) copy number 0 < 1 < 2. Furthermore, the carriers of minor alleles of rs2516839 (C) and rs1556259 (G) of *USF1* gene had decreased *USF1* expression in atherosclerotic plaques (P = 0.028 and 0.08, respectively) as compared to non-carriers. The genetic variation in *USF1* influence *USF1* transcript expression in advanced atherosclerosis and regulates levels and metabolism of circulating apoB and apoB-containing lipoprotein particles in sex-dependent manner, but is not a major determinant of early markers of atherosclerosis.

The upstream transcription factor 1 (USF1) is a ubiquitously expressed transcription factor regulating transcription of many genes from lipid and glucose metabolism pathways^1^. The USF1 contains a helix-loop-helix motif, which binds an E-box motif in the promoter region of its target genes and leads to transcription activation and/or enhanced gene expression^2^. There are very few data regarding USF1 transcript levels in human tissues. In two studies of adipose tissue, USF1 transcript levels did not differ in relation to USF1 alleles^1^,^3^. According to our knowledge *USF1* expression or the effect of its genetic variation on gene expression in artery wall has not been reported in any previous investigations.

The *USF1* gene is localized on chromosome 1q23, consists of 11 exons and extends to 6.73 kb (National Centre for Biotechnology (NCBI), gene ID: 7391). Initially this gene was localized in 1998^4^ and then identified as the first familial combined hyperlipidemia (FCHL) gene in rare Finnish pedigree with multiple affected individuals having a greatly increased risk for cardiovascular disease (CVD)^1^. This finding was rapidly replicated in Mexican families^5^. Since then *USF1* and its genetic variation has also been associated with the metabolic syndrome and type II diabetes^6^,^7^,^8^. In earlier studies we showed that some of the *USF1* gene variants are associated with the surface area of atherosclerotic lesions measured directly from coronaries after autopsy in men^9^ and reported that two *USF1* SNPs (rs3737787 and rs2516838) and *USF1* haplotype are associated with carotid intima-media thickness (cIMT)^10^.

In a study with two large prospective Finnish cohorts, the effect of *USF1* allelic variants on CVD risk and overall mortality was seen in females only^11^. Given the differences in CVD event rate, life-expectancy and mortality between men and women, the gender-specific effects of *USF1* are of obvious interest. Another study performed using mouse models showed that over-expression of human *USF1* in mice influenced metabolic trait phenotypes in sex-dependent manner^12^. There are also earlier existing sex-specific results concerning *USF1* polymorphisms and lipids parameters^1^,^13^,^14^. Any difference in the *USF1* gene effects on CVD risk factors or lipoprotein metabolisms between men and women may provide further valuable insight into the biology of the increased susceptibility of women to CVDs. These previous results give justified rationale for additional sex-specific analyses.

Early indices of atherosclerosis namely cIMT, carotid artery distensibility (Cdist), and brachial artery flow-mediated dilatation (FMD) have all been shown to predict future cardiovascular events^15^,^16^,^17^,^18^,^19^,^20^. The previous reports of *USF1* alleles on these markers are inconclusive in the sex-specific effects^10^.

In the present study, we used the ongoing prospective Cardiovascular Risk in Young Finns Study (YFS) follow-up (2001 and 2007) and Tampere Vascular Study (TVS) materials^21^,^22^,^23^ in order to investigate the effects of *USF1* gene allelic variants on serum indices of lipoprotein metabolism, early markers of asymptomatic atherosclerosis and their changes during six years follow-up separately in men and women. Since the direct contribution of these *USF1* gene variants on the gene function in artery wall tissues has not been previously tested we also investigated the *cis*-regulatory role of studied *USF1* alleles in artery wall tissues.

## Results

### Characteristics of YFS and TVS subjects

Table 1 shows the characteristics of 1608 study subjects in YFS in 2001. Overall, men had more unfavorable cardiovascular risk factor profiles compared with women. In addition, men had higher cIMT values, lower brachial artery FMD, as well as lower Cdist.

Supplementary Table 1 shows the clinical characteristics of 91 patients in TVS. Case and control subjects had similar risk factor profiles with an exception that the cases had lower BMI than the controls.

### *USF1* polymorphisms and haplotypes in YFS

The *USF1* allele and haplotype frequencies together with the six studied SNPs are shown in Table 2. A total of five common *USF1* haplotypes were identified, accounting for 97.9% of all variation in the *USF1* gene. There were no statistically significant differences in haplotype frequencies between men and women (data not shown). All *USF1* genotype distributions were in accordance with the Hardy-Weinberg equilibrium for the entire population and the subgroups divided by sex (data not shown).

### *USF1* polymorphisms, haplotypes and their sex interactions with serum lipid and apolipoprotein measurements

There were statistically significant genotype (rs2516839, rs1556259) and haplotype (H3) differences by sex interaction in relation to serum total- and LDL-cholesterol as well as apoB levels in 2007 (Table 3). In females, serum total-, LDL-cholesterol and apoB levels increased gradually according to *USF1* rs2516839 genotype TT < TC < CC and rs1556259 AA < AG < GG as well as according to *USF1* haplotype 3 (H3, GCCCGG) copy number 0 < 1 < 2, constantly in both 2001 and 2007 (Table 3). There was a similar trend for both years 2001 and 2007 for the effect of these *USF1* polymorphisms and haplotypes, although the trend tended to be statistically stronger and consistent in 2007 at aged 30–45, when the subjects were an average 6-year older than in 2001 (aged 24–39 years) (Table 3).

There were also other associations between studied *USF1* polymorphisms, haplotypes and some other classical lipid markers in 2001 and in 2007 (see Supplementary Tables 2–5), but these associations were not as consistent as they were for total-, LDL-cholesterol and apoB levels. Therefore, this study was focused on more detailed second stage analyses (by using proton-NMR spectroscopy) on mainly to those lipoprotein subclass measures with most consistent genetic associations in a sex-specific manner.

In females, both rs2516839 and rs1556259 genotypes associated significantly with the longitudinal change from 2001 to 2007 in LDL cholesterol values (P = 0.044 and P = 0.009, respectively, repeated-measurement RANCOVA main effect for genotype, age and BMI at 2001 as covariates). Similarly, the *USF1* rs1556259 genotypes associated significantly with the longitudinal change in total cholesterol levels (P = 0.021). The females carrying the haplotype 3 had significantly higher total- and LDL cholesterol concentrations throughout the six-year follow-up period compared with noncarriers of this haplotype (P = 0.008 and P = 0.006, respectively). These effects were sex-specific and were not found in males.

### *USF1* polymorphisms, haplotypes and lipid metabolism at subclass level in women

**Figure 1:**
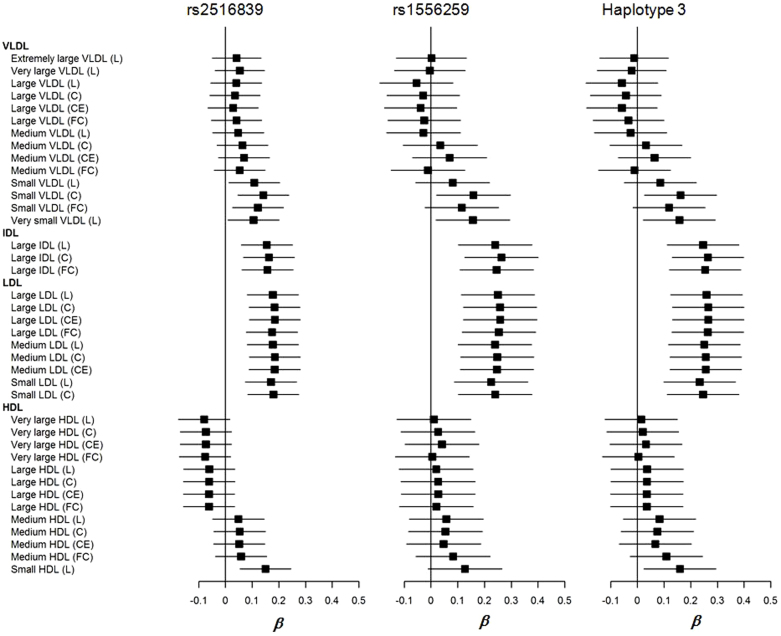
Forest plots for the associations of *USF1* polymorphisms (rs2516839, rs1556259,), haplotype 3 and serum lipoprotein subclass cholesterol fractions (total cholesterol, cholesterol ester, and free cholesterol when available) and total lipid concentrations in women in 2007. Plots show the association estimates (β) and 95% confidence intervals for the lipoprotein subclass levels presented as bars. C, total cholesterol; CE, cholesterol ester; FC, free cholesterol; IDL, intermediate-density lipoprotein; HDL, high-density lipoprotein; L, total lipids; LDL, low-density lipoprotein; VLDL, very-low-density lipoprotein.

To avoid unnecessary multiple testing the three most significant genetic markers associated with classical lipids (results from above), rs2516839, rs1556259 and haplotype H3 were selected for more detailed and focused two stage statistical analysis in relation to selected indices of cholesterol and lipid metabolism over different lipoprotein subclasses in women. We investigated the effects of *USF1* genotypes and haplotypes over serum lipoprotein subclass cholesterol fractions (total cholesterol, cholesterol ester, and free cholesterol when available) and total lipid concentrations in women. We show the associations between the above parameters and three genetic markers in women in 2007 in Figure 1. There were significant genotype and haplotype effects on the total cholesterol level of small VLDL, total lipid level of very small VLDL, total cholesterol and lipid levels and free cholesterol level of IDL, and all the three subclasses of LDL in 2007 (Figure 1 and Supplementary Table 6). These results were replicable and there was a similar trend for both follow-up years 2001 and 2007 for the effect of these polymorphisms and haplotypes, although the trend tended to be statistically stronger in 2007 at aged 30–45 (Figure 1 and Supplementary Table 6) than in 2001 (aged 24–39 years) (Supplementary Table 7). These genotype and haplotype effects are consistent with the results from the classical lipid measurements.

### *USF1* polymorphisms, haplotypes, and markers of subclinical atherosclerosis

In females, the *USF1* rs2516839 genotypes associated significantly with the cIMT in longitudinal analysis (P = 0.012, repeated-measurement ANOVA main effect for genotype). Those with CC genotype had highest cIMT in both 2001 and 2007. However, this effect disappeared after adjusting for classical risk factors for CAD and again was not seen in males. We did not find any significant differences in cIMT, brachial artery FMD, or Cdist between other *USF1* SNP genotype groups, haplotype groups in longitudinal analyses either among men or among women (data not shown).

### *USF1* expression in the atherosclerotic tissue

We compared the *USF1* mRNA expression examined with GWEA between atherosclerotic plaque samples and non-atherosclerotic internal thoracic arteries, as well as *USF1* expression between different vessel types. The *USF1* expression level (isoform 2) was significantly lower in atherosclerotic plaque specimens (N = 68) than in control tissue (N = 23, P = 0.049, Mann-Whitney U test). The *USF1* gene expression was significantly reduced in the carotid plaques (N = 29, P = 0.05, Mann-Whitney U test), but not in the femoral plaques (N = 24, P = 0.10, Mann-Whitney U test) and aorta (N = 15, P = 0.32, Mann-Whitney U test) although they followed the same trend. There was no difference in the USF1 expression between women and men.

### Relation of *USF1* polymorphisms and haplotypes to expression of *USF1* and known *USF1* target genes

Sixty-nine subjects (aged 40–91 years, males 71%) had complete data concerning the six *USF1* SNPs and gene expression data. The six studied *USF1* SNPs formed five major haplotypes. These haplotypes accounted for 97% of all variation in the *USF1* gene. Because no interaction between the SNP or haplotype and subject status (case vs. control) was found to be significant, we proceeded to analyze the effect of the haplotypes or SNPs within all samples in order to increase the statistical power. We found that minor homozygotes (CC and GG carriers) of both SNPs, rs2516839 and rs1556259, had lower *USF1* expression than the T allele and A allele carriers (P = 0.028 and P = 0.08, respectively, Mann-Whitney U test). The GCC**CG**G haplotype was the only haplotype carrying both the C allele of the rs2516839 polymorphism and G allele of the rs1556259 polymorphism. The carriers of the above haplotype tended to have lower *USF1* expression than the non-carriers (P = 0.06, Mann-Whitney U test).

Of the 47 known *USF1* target genes^24^,^25^, the chemokine (C-X-C motif) receptor 4 (CXCR4) was up-regulated in CC carriers of rs2516839 (P = 0.013, Mann-Whitney U test) and haplotype GCC**CG**G carriers (P = 0.04, Mann-Whitney U test). The hemoglobin beta (HBB) was down-regulated in CC carriers of rs2516839 (P = 0.01, Mann-Whitney U test) and haplotype GCC**CG**G carriers (P = 0.05, Mann-Whitney U test).

## Discussion

We found a female-specific association of *USF1* variants and haplotype with serum levels of both total lipids and lipoprotein subclasses in YFS. Furthermore, we found the association of these *USF1* variants and haplotype with *USF1* expression in the carotid artery plaque in TVS. To our knowledge, this study is the first showing that the *USF1* gene expression is down-regulated in atherosclerotic lesions and *USF1* variants (SNP rs2516839 and rs1556259) are associated with the down-regulation. The results of the present study imply that *USF1* variants are likely to have causal effects due to the genetic influence of the variants on gene expression.

The biological importance of the *USF1* gene has been implied in previous studies, which were mostly conducted on study subjects with specific selection criteria, such as presence of FCHL^1^,^5^, CVD^14^, diabetes^7^,^26^, metabolic syndrome^26^ or obesity^27^. There were little direct data from *in vivo* studies as to USF1 transcript levels in human tissues. In two studies of adipose tissue, USF1 transcript levels did not differ in relation to USF1 alleles^1^,^3^. One study showed clear sex-related differences in trait expression in mouse models and the gene expression patterns were also notably different between the sexes^12^. Our results are consistent with studies done using female mice, in which the enrichment for metabolism-related categories was demonstrated^12^.

The association between the *USF1* variants and lipid levels are inconsistent with previous publications. Our findings are in agreement with a population based data set from the Finns that the minor allele (C) of rs2516839 was associated with increased lipid values among study subjects with cardiovascular disease^11^. Contradictory to studies where the common allele T of rs2516839 associated with a more unfavorable risk profile^6^,^26^,^28^,^29^, the minor allele C of rs2516839 of our sample associated with increased LDL cholesterol levels. The major alleles of rs2516839 and rs1556259 were associated with increased triglycerides and lower HDL cholesterol levels in the Australian subjects with documented CAD^28^. The common allele of the SNP associated with increased cholesterol and triglyceride levels also in Utah families ascertained for type 2 diabetes mellitus^29^. In another study, the CC genotype of SNP rs2516839 showed suggestive association with decreased risk of metabolic syndrome in Chinese hospital cases^26^. The minor homozygotes of rs1556259 had lowest LDL cholesterol level in women^6^. However, in these studies, the effect of *USF1* was more pronounced in subjects already diagnosed with CAD, FCHL, diabetes and metabolic syndrome. In our study, the subjects are healthy young Finns which might explain the lack of association of *USF1* SNPs markers with lipid measures. The discrepancies regarding the minor allele of rs2516839 and rs1556259 in lipid levels in our study and other studies may be explained with the complicated interactions between *USF1* variants and age (healthy and quite young in our study), sex, other genes, and environmental factors. At the moment there were no functional studies available that could explain these differences. Thus, further functional studies of the effects of rs2516839 as well as other *USF1* variants are needed to solve this discrepancy.

We also found an association between *USF1* rs2516839 and cIMT during six years follow-up in female although this effect disappeared after adjusting for classical risk factors of CAD. The carriers of minor allele of rs2516839 had higher mean cIMT values. This is in agreement with the same allele carriers having higher LDL cholesterol levels. The sex-related differences observed in our study are in line with previous studies reporting that *USF1* variants associated with CVD^11^,^30^ and mortality^11^ among women. Contrary to the previous studies^1^,^9^,^14^, our female subjects showed stronger associations than males, and the effects of rs2516839 and rs1556259 were significant only in the female subset. In a single study that included only males^9^, the *USF1* risk allele identified was different from the risk allele segregating in our study. In 700 Finnish middle-aged men (the Helsinki Sudden Death Study, HSDS)^9^, the risk for advanced atherosclerotic plaques, calcification, and sudden cardiac death was associated with the common allele of rs2516839, whereas in the present study the minor allele of rs2516839, was associated with elevated LDL cholesterol level and cIMT. Interestingly, in female U.S. Whites with CAD, the rare allele was associated with risk, whereas in males the common allele of rs3737787 conferred risk^13^. Consistent with human studies, the sex specificity was also observed in a mouse model^12^. The molecular mechanisms underlying our sex-specific allelic difference are unknown. The hormonal factors may contribute to the differences.

USF1 is a ubiquitous transcription factor that regulates the expression of many genes involved in lipid metabolism, immune response, endothelial function and aging. It could contribute to the development of atherosclerosis and its complications through many different pathways. In our study, we did not find significant *USF1* genotype difference of gene expression in most of the previously identified USF1 target genes except CXCR4 and HBB. Previous studies have reported that the CXCR4 levels are increased in patients with heart failure^31^ and that myocardial CXCR4 levels are increased 5-fold in myocardium subjected to ischemic injury, compared with levels in non-injured myocardium in the same heart^32^. Other studies have also reported that the hemoglobin levels are independently associated with increased risk for new cardiac events^33^,^34^. Further studies are needed for understanding the mechanism behind our findings between the *USF1* variants and the up-regulated CXCR4 and down-regulated HBB expression.

We did not find any significant difference of USF1 expression between male and female in TVS study. These expression results in TVS samples should be interpreted with caution due to small sample size and low number of women, and thus possible lack of statistical power. It would be interesting to repeat the observation with larger numbers of subjects.

In conclusion, our findings support the role of *USF1* variants and haplotypes in lipid metabolism in gender-dependent manner and establish a new association of *USF1* variants with gene expression in carotid arteries. Further research is needed on the associations between the *USF1* SNPs, haplotypes and cardiovascular end points.

## Methods

### Study subjects

The YFS is an ongoing five-center, prospective cohort study of atherosclerosis risk factors underlying cardiovascular disease in children and young adults. Details of the study design have been presented elsewhere^22^. In short, the study was launched in 1980 and included 3,596 children and adolescents aged 3 to 18 years. In 2001, a total of 2,283 participants aged 24–39 years were re-examined, and in 2007, we examined 2,204 subjects aged 30–45 years. The present study included 1,608 subjects for whom complete data on lipids and cIMT in both year 2001 and 2007 were available (female, N = 902; male, N = 706).

All subjects gave a written informed consent in 2001 and 2007. The study was approved by the local ethics committees. The methods were carried out in accordance with the approved guidelines. More detailed information about the cohorts and the follow-up procedures can be found in the cohort descriptions of the Project (http://vanha.med.utu.fi/cardio/youngfinnsstudy/index.html).

### Clinical characteristics

Weight and height were measured, and body mass index (BMI) was calculated. Blood pressure was measured with a random zero sphygmomanometer. The average of three measurements was used in the analyses. Information on smoking, alcohol consumption, and physical activity was obtained with a questionnaire. Those smoking on daily basis were defined as smokers.

### Biochemical analyses

In 2001 and 2007, venous samples were taken after the subject had fasted for 12 hours. Serum total cholesterol levels were measured by the enzymatic cholesterol esterase – cholesterol oxidase method (Cholesterol reagent, Olympus, Ireland). The same reagent was used for estimating HDL-cholesterol levels after precipitation of apoB-containing lipoproteins with dextran sulfate-Mg^2+^. LDL-cholesterol was estimated by the Friedewald formula^35^ in subjects with triglycerides levels <4.0 mmol/L. The serum triglyceride concentration was assayed using the enzymatic glycerol kinase-glycerol phosphate oxidase method (Triglyceride reagent, Olympus). Serum glucose concentration was determined by the enzymatic hexokinase method (Glucose reagent, Olympus). Apolipoprotein A1 (ApoA1) and B were analysed immunoturbidometrically (Orion Diagnostica, Espoo, Finland). The above mentioned analyses were all performed on an AU400-analyzer (Olympus, Japan). Serum insulin concentration was determined by a microparticle enzyme immunoassay (IMx insulin reagent, Abbott Diagnostics, USA) on an IMx instrument (Abbott). The method has been described in more detail elsewhere^36^. Fasting plasma high sensitive C-reactive protein (CRP) concentrations were analyzed by means of latex turbidometric immunoassay (Wako Chemicals GmbH, Neuss, Germany).

### Measurements of carotid artery IMT and brachial artery FMD

Ultrasound examinations were performed using Sequoia 512 ultrasound mainframes (Acuson, CA, USA) with 13.0 MHz linear array transducers, as described previously in detail^37^. In short, to measure carotid IMT, the image was focused on the posterior (far) wall of the left carotid artery. A minimum of four measurements of the common carotid far wall was taken approximately 10 mm proximal to the bifurcation to derive mean and maximal cIMT values. To evaluate brachial artery FMD, the left brachial artery diameter was measured both at rest and during reactive hyperemia, as described previously^38^. The vessel diameter in scans after reactive hyperemia was expressed as a percentage relative to the resting scan value (%). To assess carotid artery elasticity indices, the best–quality cardiac cycle was selected from the images and manually analyzed to measure systolic and diastolic common carotid diameters. The carotid distensibility (Cdist) was then calculated using ultrasound and concomitant brachial blood pressure measurements, as described previously^39^. These analyses were performed for YFS subjects in 2001 and in 2007.

### Lipoprotein subclass analysis by proton-NMR spectroscopy

Concentrations of lipoprotein subclasses as well as their cholesterol content were analyzed by proton NMR spectroscopy in native serum samples as described previously^40^. These serum subclasses were classified as follows: chylomicrons and extremely large VLDL particles (average particle diameter at least 75 nm); five different VLDL subclasses: very large VLDL (average particle diameter of 64.0 nm), large VLDL (53.5 nm), medium VLDL (44.5 nm), small VLDL (36.8 nm), and very small VLDL (31.3 nm); intermediate-density lipoprotein (IDL) (28.6 nm); three LDL subclasses: large LDL (25.5 nm), medium LDL (23.0 nm), and small LDL (18.7 nm); and four HDL subclasses: very large HDL (14.3 nm), large HDL (12.1 nm), medium HDL (10.9 nm), and small HDL (8.7 nm)^40^. This methodology has recently been applied in various extensive epidemiological and genetics studies^41^,^42^,^43^ with consistent findings with respect to lipoprotein genetics^44^.

### DNA extraction and genotyping of the *USF1* polymorphisms

For subjects in YFS, DNA was extracted from peripheral blood leukocytes using a commercially available kit (Qiagen Inc, Hilden, Germany) in 2001. DNA samples were genotyped by employing the 5′ nuclease assay for allelic discrimination, using the ABI Prism 7900 HT Sequence Detection System (Applied Biosystems, Foster City, CA). PCR reaction containing genomic DNA, 2 × TaqMan Universal PCR Master Mix, 900 nM of each primer, and 200 nM of each probe was performed in 384-well plates according to standard protocol in a total volume of 5 μl. Water controls and known control samples were run in parallel with unknown samples. After cycling, end-point fluorescence was measured, and *USF1* genotypes (rs3737787, rs2073658, rs2516838, rs10908821, rs2516839, rs1556259, rs2774279, rs2774276) calling was carried out by the allelic discrimination analysis module. The eight SNPs were selected from the HapMap database and SeattleSNPs database and previous publications. Two of the SNPs selected in the preliminary phase, rs2073658 and rs2774279, were left out from further analyses because they were in almost complete linkage disequilibrium with rs3737787 and rs2516838, respectively. The linkage between rs2516838 and rs2516839 was weak (r^2^ = 0.22).

In TVS, genomic DNA was extracted from peripheral blood leukocytes using QIAamp DNA Blood Minikit and automated biorobot M48 extraction (Qiagen, Hilden, Germany). Whole genome genotyping using the Illumina HumanHap660W-Quad BeadChip (Illumina, Inc., San Diego, CA, USA) was carried out from TVS samples according to manufactures recommendation as described in detail in supplementary methods.

### Vascular sample collection, RNA isolation, expression analysis and quality control

The atherosclerotic plaque samples (male, N = 46; female, N = 22) and non-atherosclerotic control samples (male, N = 20; female, N = 3, left internal thoracic arteries, LITA) used in this study were collected as part of ongoing TVS^21^,^23^,^45^,^46^,^47^. The study was approved by the Ethics Committee of Tampere University Hospital, and the study subjects gave their informed consent. The clinical characteristics of TVS subjects are shown in supplementary Table 1 and details of sample collection in supplementary methods. All open vascular surgical procedures were performed at the Division of Vascular Surgery and control samples collected at Heart Center, Tampere University Hospital. The study was approved by the Ethics Committee of Tampere University Hospital, and the study subjects gave their informed consent. The vascular samples were classified according to recommendations of American Heart Association (AHA)^48^ by experienced pathologist. All the internal thoracic artery samples that were used as controls were verified to be microscopically healthy.

In TVS, vascular endarterectomy samples constituting the intima and inner media from carotid, femoral and aortic regions were obtained. RNA isolation, genome-wide expression analysis, RNA quality control of these atherosclerotic plaque and non-atherosclerotic control tissue samples, was performed as previously described^21^,^23^,^45^,^46^,^47^ (see details in supplementary methods).

### Statistical analyses

Statistical analyses were performed using the PASW Statistics 18. Non–normally distributed triglycerides, insulin, and CRP concentrations were log_10_-transformed before the analyses, but the results are expressed as crude. Data are presented as mean ± SD, unless otherwise stated. The characteristics of the study subjects were compared with the *t* test for continuous variables. Categorical variables were compared with the χ^2^–test, which was also used to test the genotype frequencies under Hardy–Weinberg equilibrium. In order to study the possible association between *USF1* gene polymorphisms and risk factors and subclinical markers of atherosclerosis, we applied analysis of variance (ANOVA) and analysis of covariance (ANCOVA). The non-parametric Mann-Whitney U test was used for comparison of gene expression between atherosclerotic and control tissues, as well as between different genotype and haplotype groups.

Frequencies of the most common haplotypes and the most probable haplotypes for each study subject were determined using the PHASE program (Version 2.0.2)^49^. To study the effect of haplotypes, we divided the population into carriers (one copy and two copies of haplotype) and non-carriers or into three groups with 0, 1, 2 copy of haplotype.

To avoid redundant multiple testing in YFS, the statistical analyses were done in separate stages. We first did sex-by-genotype (or -by-haplotype) interaction analyses in relation to conventional lipids and apolipoproteins by two-way ANOVA. If significant interaction was found, we stratified the subjects by sex in further analyses. As hypothesized, sex-specific interactions in relation to serum total- and LDL-cholesterol and apoB were found and the *USF1* genotype/haplotype related results were statistically significant in women only. In the second stage of statistical analysis, we investigated in women the effects of *USF1* genotypes and haplotypes over serum lipoprotein subclass cholesterol fractions (total cholesterol, cholesterol ester, and free cholesterol when available) and total lipid concentrations.

The subclass measures were inverse normal transformed to achieve approximate normality. Associations of *USF1* SNPs and haplotypes with lipoprotein subclass measures were tested using univariate additive genetic model. Statistical analyses were performed using the R Statistical package v. 2.11.1 (http://www.r-project.org).

P–value < 0.05 was considered nominally significant. For Table 3, we calculated false discovery rate (FDR) multiple correction calculations in the women strata assuming there were 18 independent tests (2 SNPs and 1 haplotype, 2 time points, 3 lipid measures), using the calculation below and assuming an FDR value of < 0.05 was acceptable.

FDR = p-value × number of tests/p – value rank

Therefore, P ≤ 0.015 in the women strata were considered statistically significant after correcting for multiple testing.

## Author Contributions

Y.-M.F. participated in the study design, performed the most of the data analyses and drafted the manuscript. J.H., A.C. were responsible for part of data analyses. N.O., A.M., M.T., J.P.S. contributed to vascular sample collection and associated data collection. M.L., E.R. did the vascular sample handling and RNA extraction. M.J., J.M., J.V., O.T.R., N.H.K., M.K., were involved in the initial Young Finns Study and the writing. L.P.L., I.S. did part of data analyses and prepared Figure 1. N.K., T.I. contributed to expression analysis. A.J.K., P.S., M.A.K. designed and performed the NMR analyses. T.L. contributed to measurements of carotid artery IMT and brachial artery FMD. R.L., T.L. participated in the study design and revised the manuscript critically. All investigators contributed to the writing of the manuscript. All authors reviewed the manuscript.

## Supplementary Material

Supplementary Information Supplementary Tables and Methods

## Figures and Tables

**Table 1:** Characteristics of the Cardiovascular Risk in Young Finns Study population in 2001

Variable	men	women
No. of subjects	706	902
Age, years	31.9 ± 5.0	31.9 ± 4.9
Body mass index, kg/m^2^	25.6 ± 3.8	24.2 ± 4.3
Systolic blood pressure, mm Hg	129 ± 14	116 ± 12
Diastolic blood pressure, mm Hg	75 ± 9	71 ± 9
Total cholesterol, mmol/L	5.22 ± 0.98	5.03 ± 0.87
LDL cholesterol, mmol/L	3.42 ± 0.88	3.13 ± 0.75
HDL cholesterol, mmol/L	1.17 ± 0.27	1.40 ± 0.30
Triglycerides, mmol/L	1.42 ± 0.82	1.13 ± 0.53
Apolipoprotein A1, g/L	1.40 ± 0.21	1.56 ± 0.26
Apolipoprotein B, g/L	1.12 ± 0.26	0.99 ± 0.23
Glucose, mmol/L	5.21 ± 0.87	4.90 ± 0.72
Insulin, mU/L	7.46 ± 5.88	7.61 ± 5.63
CRP, mg/L	1.43 ± 3.42	2.18 ± 4.33
Daily smoking, %	46.6	36.9
IMT 2001, mm	0.59 ± 0.10	0.57 ± 0.09
FMD 2001, %	6.83 ± 4.05	8.75 ± 4.49
Cdist 2001, %/10 mmHg	2.02 ± 0.67	2.33 ± 0.77

Values are mean ± SD or percentage of subjects. All comparisons (*t* tests) between men and women *P* < 0.001, except for age and insulin (*P* > 0.6).

**Table 2:** Details of the loci and haplotypes in the *USF1* gene

	Usf1s1	Usf1s8	Usf1-4530	Usf1s7	Usf1s9	Usf1-81	
dbSNP ID	rs3737787	rs2516838	rs10908821	rs2516839	rs1556259	rs2774276	
Allele	G/A	C/G	C/G	T/C	A/G	C/G	
MAF	0.357	0.271	0.136	0.371	0.131	0.233	
haplotype							Frequency (%)
H1	A	C	C	T	A	G	35.4
H2	G	G	C	T	A	G	26.3
H3	G	C	C	C	G	G	13.2
H4	G	C	G	C	A	C	13.1
H5	G	C	C	C	A	C	10.5

MAF, minor allele frequency. The minor allele of each SNP is underlined.

**Table 3:** The association of serum total- and LDL-cholesterol and apolipoprotein B levels with *USF1* genotypes and haplotype 3 (H3) according to gender and follow-up year. The Cardiovascular Risk in Young Finns Study

Follow-up Year	SNP or haplotype	Genotypeor copy of haplotype	N (women)	Mean ± SD	P	N (men)	Mean ± SD	P	N (all)	Mean ± SD	P
Total cholesterol (mmol/L)											
2001	rs2516839	TT	349	5.01 ± 0.85	0.67	282	5.27 ± 1.00	0.536	631	5.12 ± 0.93	0.82
		TC	419	5.04 ± 0.89		326	5.18 ± 0.94		745	5.10 ± 0.91	
		CC	123	5.09 ± 0.90		96	5.22 ± 1.05		219	5.14 ± 0.97	
	rs1556259	AA	665	5.00 ± 0.86	0.041	536	5.23 ± 0.99	0.848	1201	5.10 ± 0.93	0.335
		AG	218	5.10 ± 0.90		150	5.18 ± 0.95		368	5.13 ± 0.92	
		GG	12	5.57 ± 1.03		13	5.19 ± 0.65		25	5.37 ± 0.86	
	H3	0	670	5.00 ± 0.86	0.047	543	5.23 ± 0.99	0.882	1213	5.10 ± 0.93	0.328
		1	217	5.11 ± 0.90		148	5.18 ± 0.96		365	5.14 ± 0.92	
		2	15	5.47 ± 0.97		15	5.22 ± 0.62		30	5.34 ± 0.81	
2007	rs2516839	TT	349	4.81 ± 0.80	**0.013**	282	5.20 ± 0.91	0.392	631	4.98 ± 0.87	0.254
		TC	419	4.93 ± 0.79		326	5.10 ± 0.89		745	5.01 ± 0.84	
		CC	123	5.03 ± 0.87		96	5.18 ± 0.90		219	5.10 ± 0.89	
	rs1556259	AA	665	4.85 ± 0.80	**0.009**	536	5.16 ± 0.91	0.443	1201	4.99 ± 0.86	0.182
		AG	218	5.02 ± 0.82		150	5.07 ± 0.85		368	5.04 ± 0.83	
		GG	12	5.22 ± 0.97		13	5.31 ± 0.55		25	5.26 ± 0.77	
	H3	0	670	4.85 ± 0.80	**0.006**	543	5.17 ± 0.92	0.336	1213	4.99 ± 0.87	0.129
		1	217	5.02 ± 0.82		148	5.07 ± 0.85		365	5.04 ± 0.83	
		2	15	5.23 ± 0.93		15	5.34 ± 0.63		30	5.29 ± 0.78	
LDL cholesterol (mmol/L)											
2001	rs2516839	TT	349	3.10 ± 0.76	0.57	282	3.45 ± 0.88	0.567	631	3.26 ± 0.84	0.644
		TC	419	3.14 ± 0.73		326	3.38 ± 0.85		745	3.24 ± 0.79	
		CC	123	3.18 ± 0.78		96	3.46 ± 0.96		219	3.30 ± 0.87	
	rs1556259	AA	665	3.11 ± 0.74	**0.011**	536	3.42 ± 0.88	0.809	1201	3.25 ± 0.82	0.128
		AG	218	3.18 ± 0.76		150	3.37 ± 0.90		368	3.26 ± 0.83	
		GG	12	3.72 ± 0.88		13	3.46 ± 0.79		25	3.58 ± 0.83	
	H3	0	670	3.10 ± 0.73	**0.015**	543	3.43 ± 0.87	0.716	1213	3.25 ± 0.82	0.114
		1	217	3.19 ± 0.77		148	3.37 ± 0.90		365	3.26 ± 0.83	
		2	15	3.61 ± 0.85		15	3.51 ± 0.75		30	3.56 ± 0.79	
2007	rs2516839	TT	349	2.85 ± 0.69	**0.002**	282	3.30 ± 0.80	0.619	631	3.05 ± 0.77	0.068
		TC	419	2.96 ± 0.69		326	3.24 ± 0.79		745	3.08 ± 0.75	
		CC	123	3.10 ± 0.73		96	3.31 ± 0.79		219	3.19 ± 0.76	
	rs1556259	AA	665	2.89 ± 0.68	**0.003**	536	3.27 ± 0.79	0.276	1201	3.06 ± 0.76	0.026
		AG	218	3.05 ± 0.74		150	3.22 ± 0.78		368	3.12 ± 0.76	
		GG	12	3.30 ± 0.75		13	3.57 ± 0.56		25	3.44 ± 0.66	
	H3	0	670	2.89 ± 0.68	**0.003**	543	3.28 ± 0.80	0.160	1213	3.07 ± 0.76	0.022
		1	217	3.05 ± 0.74		148	3.21 ± 0.78		365	3.12 ± 0.76	
		2	15	3.26 ± 0.75		15	3.60 ± 0.64		30	3.43 ± 0.71	
Apolipoprotein B (g/L)											
2001	rs2516839	TT	349	0.97 ± 0.23	0.469	282	1.12 ± 0.26	0.924	631	1.04 ± 0.26	0.886
		TC	419	0.99 ± 0.23		326	1.12 ± 0.24		745	1.05 ± 0.24	
		CC	123	1.00 ± 0.23		96	1.11 ± 0.27		219	1.05 ± 0.25	
	rs1556259	AA	665	0.98 ± 0.23	0.309	536	1.12 ± 0.26	0.804	1201	1.04 ± 0.25	0.352
		AG	218	0.99 ± 0.24		150	1.11 ± 0.24		368	1.04 ± 0.25	
		GG	12	1.08 ± 0.25		13	1.15 ± 0.21		25	1.11 ± 0.23	
	H3	0	670	0.98 ± 0.23	0.33	543	1.12 ± 0.26	0.781	1213	1.04 ± 0.25	0.36
		1	217	1.00 ± 0.24		148	1.11 ± 0.25		365	1.04 ± 0.25	
		2	15	1.06 ± 0.23		15	1.16 ± 0.20		30	1.11 ± 0.22	
2007	rs2516839	TT	349	0.91 ± 0.22	**0.015**	282	1.11 ± 0.25	0.222	631	1.00 ± 0.25	0.46
		TC	419	0.94 ± 0.22		326	1.08 ± 0.24		745	1.00 ± 0.24	
		CC	123	0.98 ± 0.24		96	1.08 ± 0.25		219	1.02 ± 0.25	
	rs1556259	AA	665	0.93 ± 0.22	0.049	536	1.10 ± 0.25	0.638	1201	1.00 ± 0.25	0.388
		AG	218	0.96 ± 0.23		150	1.08 ± 0.23		368	1.00 ± 0.24	
		GG	12	1.05 ± 0.24		13	1.09 ± 0.20		25	1.07 ± 0.22	
	H3	0	670	0.93 ± 0.22	0.055	543	1.10 ± 0.25	0.621	1213	1.00 ± 0.25	0.421
		1	217	0.96 ± 0.23		148	1.08 ± 0.23		365	1.00 ± 0.24	
		2	15	1.03 ± 0.23		15	1.09 ± 0.19		30	1.06 ± 0.21	

Values are mean ± SD. ANOVA. Significant values within the women strata after correcting for multiple testing (P ≤ 0.015) are in bold.
