# Supplementary material for: Upstream Transcription Factor 1 (USF1) allelic variants regulate lipoprotein metabolism in women and USF1 expression in atherosclerotic plaque
Source: Sci Rep. 2014 Apr 11;4:4650. doi: 10.1038/srep04650 (PMC3983598; doi:10.1038/srep04650)
Supplement: Supplementary Information — Supplementary Tables and Methods [file srep04650-s1.pdf]

Upstream Transcription Factor 1 (*USF1*) allelic variants regulate lipoprotein metabolism in women and USF1 expression in atherosclerotic plaque

Yue-Mei Fan[1], Jussi Hernesniemi[1], Niku Oksala[1,9], Mari Levula[1], Emma Raitoharju[1], Auni Collings[1], Nina Hutri-Kähönen[2], Markus Juonala[3], Jukka Marniemi[4], Leo-Pekka Lyytikäinen[1], Ilkka Seppälä[1], Ari Mennander[5], Matti Tarkka[5], Antti J. Kangas[6,7], Pasi Soininen[6,7], Juha Pekka Salenius[9], Norman Klopp[10], Thomas Illig[10], Tomi Laitinen[11], Mika Ala-Korpela[6,7,8,12], Reijo Laaksonen[1], Jorma Viikari[13], Mika Kähönen[14], Olli T. Raitakari[15], Terho Lehtimäki[1]

Supplementary Table 1. Characteristics of the Tampere Vascular Study subjects

Variable	Case	Control
No. of subjects	68	23
Age, years	69.7 ± 10.4	67.7 ± 9.4
Body mass index, kg/m^2	26.2 ± 3.9	28.7 ± 5.3
Systolic blood pressure, mm Hg	150 ± 31	140 ± 16
Diastolic blood pressure, mm Hg	83 ± 15	77 ± 10
Ever smoker, %	77.3	63.6
Male, %	67.6	87.0
Hypercholesterolemia, %	69.7	91.3
Hypertension, %	83.6	100
Diabetes, %	23.5	34.8

Values are mean ± SD or percentage of subjects. All comparisons (*t* test or χ^2 test when appropriate) between atherosclerotic lesions (Cases) and histological normal artery (Controls) $P>0.05$, except for BMI.

Supplementary Table 2. The association of *USF1* polymorphisms and the lipid parameters in 2001 according to gender (The Cardiovascular Risk in Young Finns Study)

SNP no.	Genotype	N (women)	Mean ± SD	P	N (men)	Mean ± SD	P	N (all)	Mean ± SD	P
**Total cholesterol (mmol/L)**										
rs3737787	GG	376	5.05 ± 0.92	0.732	271	5.21 ± 1.03	0.737	647	5.12 ± 0.97	0.994
	GA	407	5.04 ± 0.83		324	5.23 ± 0.94		731	5.12 ± 0.88	
	AA	107	4.98 ± 0.88		89	5.30 ± 0.97		196	5.12 ± 0.93	
rs2516838	CC	462	5.05 ± 0.86	0.655	358	5.22 ± 0.96	0.926	820	5.12 ± 0.91	0.956
	CG	348	5.00 ± 0.87		279	5.25 ± 1.00		627	5.11 ± 0.94	
	GG	67	5.07 ± 0.90		41	5.22 ± 1.05		108	5.13 ± 0.96	
rs10908821	CC	663	5.05 ± 0.88	0.373	522	5.25 ± 0.99	0.07	1185	5.14 ±0.93	0.22
	CG	207	5.00 ± 0.85		163	5.10 ± 0.91		370	5.04 ± 0.88	
	GG	15	4.76 ± 0.84		15	5.62 ± 1.25		30	5.19 ± 1.13	
rs2516839	TT	349	5.01 ± 0.85	0.67	282	5.27 ± 1.00	0.536	631	5.12 ± 0.93	0.82
	TC	419	5.04 ± 0.89		326	5.18 ± 0.94		745	5.10 ± 0.91	
	CC	123	5.09 ± 0.90		96	5.22 ± 1.05		219	5.14 ± 0.97	
rs1556259	AA	665	5.00 ± 0.86	**0.041**	536	5.23 ± 0.99	0.848	1201	5.10 ± 0.93	0.335
	AG	218	5.10 ± 0.90		150	5.18 ± 0.95		368	5.13 ± 0.92	
	GG	12	5.57 ± 1.03		13	5.19 ± 0.65		25	5.37 ± 0.86	
rs2774276	GG	517	5.05 ± 0.88	0.908	400	5.26 ± 0.96	0.226	917	5.14 ± 0.92	0.342
	GC	302	5.02 ± 0.86		243	5.15 ± 0.94		545	5.08 ± 0.90	
	CC	50	5.04 ± 1.03		40	5.36 ± 1.21		90	5.18 ± 1.11	
**LDL cholesterol (mmol/L)**										
rs3737787	GG	376	3.15 ± 0.79	0.768	271	3.42 ± 0.94	0.954	647	3.26 ± 0.86	0.992
	GA	407	3.13 ± 0.71		324	3.42 ± 0.85		731	3.26 ± 0.79	
	AA	107	3.09 ± 0.78		89	3.45 ± 0.79		196	3.25 ± 0.80	
rs2073658	CC	374	3.14 ± 0.79	795	272	3.42 ± 0.93	0.967	646	3.26 ± 0.86	0.991
	CT	407	3.12 ± 0.70		324	3.43 ± 0.85		731	3.26 ± 0.78	
	TT	106	3.10 ± 0.78		88	3.44 ± 0.79		194	3.25 ± 0.80	

rs2516838	CC	462	3.15 ± 0.74	0.636	358	3.43 ± 0.84	0.953	820	3.27 ± 0.79	0.924
	CG	348	3.10 ± 0.73		279	3.45 ± 0.93		627	3.25 ± 0.85	
	GG	67	3.15 ± 0.87		41	3.42 ± 0.87		108	3.25 ± 0.87	
rs10908821	CC	663	3.14 ± 0.77	0.612	522	3.44 ± 0.89	**0.047**	1185	3.27 ± 0.84	0.105
	CG	207	3.09 ± 0.70		163	3.32 ± 0.80		370	3.19 ± 0.75	
	GG	15	3.04 ± 0.72		15	3.86 ± 1.09		30	3.45 ± 1.00	
rs2774279	CC	477	3.14 ± 0.73	0.612	369	3.41 ± 0.84	0.92	846	3.26 ± 0.79	0.979
	CT	356	3.10 ± 0.75		293	3.43 ± 0.93		649	3.25 ± 0.85	
	TT	67	3.18 ± 0.85		42	3.40 ± 0.89		109	3.27 ± 0.87	
rs2774276	GG	517	3.13 ± 0.77	0.977	400	3.44 ± 0.86	0.174	917	3.27 ± 0.83	0.493
	GC	302	3.13 ± 0.71		243	3.35 ± 0.83		545	3.23 ± 0.78	
	CC	50	3.11 ± 0.83		40	3.60 ± 1.06		90	3.33 ± 0.97	
**HDL cholesterol (mmol/L)**										
rs3737787	GG	376	1.40 ± 0.30	0.26	271	1.16 ± 0.26	0.593	647	1.30 ± 0.30	0.745
	GA	407	1.41 ± 0.31		324	1.16 ± 0.27		731	1.30 ± 0.32	
	AA	107	1.36 ± 0.28		89	1.19 ± 0.29		196	1.28 ± 0.29	
rs2516838	CC	462	1.39 ± 0.31	0.33	358	1.16 ± 0.27	0.741	820	1.29 ± 0.31	0.137
	CG	348	1.39 ± 0.28		279	1.16 ± 0.26		627	1.29 ± 0.29	
	GG	67	1.45 ± 0.33		41	1.19 ± 0.31		108	1.35 ± 0.34	
rs10908821	CC	663	1.41 ± 0.30	0.059	522	1.16 ± 0.27	0.726	1185	1.30 ± 0.31	0.092
	CG	207	1.38 ± 0.30		163	1.18 ± 0.25		370	1.29 ± 0.30	
	GG	15	1.23 ± 0.26		15	1.12 ± 0.27		30	1.18 ± 0.26	
rs2516839	TT	349	1.40 ± 0.30	0.852	282	1.18 ± 0.28	0.531	631	1.30 ± 0.31	0.684
	TC	419	1.39 ± 0.30		326	1.16 ± 0.26		745	1.29 ± 0.30	
	CC	123	1.39 ± 0.32		96	1.16 ± 0.25		219	1.29 ± 0.31	
rs1556259	AA	665	1.39 ± 0.30	0.659	536	1.17 ± 0.26	0.346	1201	1.29 ± 0.30	0.338
	AG	218	1.41 ± 0.31		150	1.17 ± 0.27		368	1.31 ± 0.32	
	GG	12	1.42 ± 0.32		13	1.06 ± 0.20		25	1.23 ± 0.31	
rs2774276	GG	517	1.41 ± 0.30	0.294	400	1.17 ± 0.27	0.947	917	1.31 ± 0.31	0.338
	GC	302	1.38 ± 0.28		243	1.17 ± 0.25		545	1.28 ± 0.29	
	CC	50	1.38 ± 0.37		40	1.17 ± 0.24		90	1.29 ± 0.34	

**Triglycerides (mmol/L)**										
rs3737787	GG	376	1.14 ± 0.54	0.516	271	1.40 ± 0.73	0.777	647	1.25 ± 0.64	0.499
	GA	407	1.12 ± 0.49		324	1.43 ± 0.80		731	1.26 ± 0.66	
	AA	107	1.19 ± 0.67		89	1.47 ± 1.17		196	1.31 ± 0.94	
rs2516838	CC	462	1.14 ± 0.55	0.691	358	1.41 ± 0.91	0.774	820	1.26 ± 0.74	0.478
	CG	348	1.14 ± 0.53		279	1.45 ± 0.73		627	1.27 ± 0.64	
	GG	67	1.08 ± 0.51		41	1.36 ± 0.87		108	1.19 ± 0.68	
rs10908821	CC	663	1.12 ± 0.52	0.425	522	1.44 ± 0.88	0.458	1185	1.26 ± 0.72	0.967
	CG	207	1.17 ± 0.58		163	1.35 ± 0.63		370	1.25 ± 0.61	
	GG	15	1.10 ± 0.56		15	1.44 ± 0.59		30	1.27 ± 0.59	
rs2516839	TT	349	1.12 ± 0.56	0.832	282	1.43 ± 0.91	0.598	631	1.26 ± 0.75	0.886
	TC	419	1.13 ± 0.51		326	1.43 ± 0.79		745	1.26 ± 0.66	
	CC	123	1.16 ± 0.56		96	1.34 ± 0.67		219	1.24 ± 0.61	
rs1556259	AA	665	1.14 ± 0.55	0.583	536	1.42 ± 0.82	0.934	1201	1.26 ± 0.70	0.94
	AG	218	1.13 ± 0.50		150	1.42 ± 0.82		368	1.25 ± 0.66	
	GG	12	0.98 ± 0.35		13	1.50 ± 1.14		25	1.25 ± 0.88	
rs2774276	GG	517	1.12 ± 0.53	0.391	400	1.45 ± 0.91	0.596	917	1.26 ± 0.74	0.957
	GC	302	1.13 ± 0.54		243	1.40 ± 0.72		545	1.25 ± 0.64	
	CC	50	1.23 ± 0.58		40	1.33 ± 0.54		90	1.27 ± 0.56	
**Apolipoprotein B (g/L)**										
rs3737787	GG	376	1.00 ± 0.23	0.659	271	1.12 ± 0.27	0.952	647	1.05 ± 0.26	0.942
	GA	407	0.98 ± 0.22		324	1.12 ± 0.25		731	1.04 ± 0.24	
	AA	107	0.99 ± 0.24		89	1.13 ± 0.26		196	1.05 ± 0.26	
rs2516838	CC	462	0.99 ± 0.23	0.902	358	1.12 ± 0.25	0.843	820	1.05 ± 0.25	0.817
	CG	348	0.98 ± 0.23		279	1.13 ± 0.25		627	1.05 ± 0.25	
	GG	67	0.98 ± 0.25		41	1.11 ± 0.31		108	1.03 ± 0.28	
rs10908821	CC	663	0.99 ± 0.23	0.947	522	1.12 ± 0.26	0.129	1185	1.05 ± 0.25	0.303
	CG	207	0.98 ± 0.22		163	1.09 ± 0.24		370	1.03 ± 0.23	
	GG	15	0.97 ± 0.23		15	1.20 ± 0.34		30	1.09 ± 0.31	
rs2516839	TT	349	0.97 ± 0.23	0.469	282	1.12 ± 0.26	0.924	631	1.04 ± 0.26	0.886
	TC	419	0.99 ± 0.23		326	1.12 ± 0.24		745	1.05 ± 0.24	

	CC	123	1.00 ± 0.23		96	1.11 ± 0.27		219	1.05 ± 0.25	
rs1556259	AA	665	0.98 ± 0.23	0.309	536	1.12 ± 0.26	0.804	1201	1.04 ± 0.25	0.352
	AG	218	0.99 ± 0.24		150	1.11 ± 0.24		368	1.04 ± 0.25	
	GG	12	1.08 ± 0.25		13	1.15 ± 0.21		25	1.11 ± 0.23	
rs2774276	GG	517	0.98 ± 0.24	0.767	400	1.12 ± 0.26	0.601	917	1.04 ± 0.26	0.866
	GC	302	0.99 ± 0.22		243	1.11 ± 0.25		545	1.04 ± 0.24	
	CC	50	0.99 ± 0.26		40	1.14 ± 0.30		90	1.06 ± 0.29	

Supplementary Table 3. The association of *USF1* haplotypes and lipid parameters in 2001 according to gender (The Cardiovascular Risk in Young Finns Study)

Haplotype no.	copy	N (women)	Mean ± SD	P	N (men)	Mean ± SD	P	N (all)	Mean ± SD	P
**Total cholesterol (mmol/L)**										
H1	0	386	5.05 ± 0.92	0.62	283	5.21 ± 1.01	0.854	669	5.12 ± 0.96	0.977
	1	409	5.04 ± 0.82		333	5.22 ± 0.94		742	5.12 ± 0.88	
	2	107	4.96 ± 0.87		90	5.27 ± 1.00		197	5.10 ± 0.94	
H2	0	490	5.05 ± 0.88	0.434	380	5.20 ± 0.96	0.857	870	5.12 ± 0.92	0.868
	1	346	4.99 ± 0.86		286	5.24 ± 0.99		632	5.11 ± 0.93	
	2	66	5.12 ± 0.89		40	5.22 ± 1.06		106	5.16 ± 0.96	
H3	0	670	5.00 ± 0.86	**0.047**	543	5.23 ± 0.99	0.882	1213	5.10 ± 0.93	0.328
	1	217	5.11 ± 0.90		148	5.18 ± 0.96		365	5.14 ± 0.92	
	2	15	5.47 ± 0.97		15	5.22 ± 0.62		30	5.34 ± 0.81	
H4	0	681	5.06 ± 0.88	0.212	530	5.25 ± 0.98	0.067	1211	5.14 ± 0.93	0.123
	1	207	4.98 ± 0.84		161	5.10 ± 0.92		368	5.03 ± 0.88	
	2	14	4.73 ± 0.86		15	5.62 ± 1.25		29	5.19 ± 1.15	
H5	0	727	5.02 ± 0.87	0.263	568	5.22 ± 0.95	0.842	1295	5.11 ± 0.91	0.605
	1	165	5.07 ± 0.86		135	5.23 ± 1.07		300	5.14 ± 0.96	
	2	10	5.44 ± 1.22		3	4.90 ± 1.00		13	5.32 ± 1.16	
**LDL cholesterol (mmol/L)**										
H1	0	386	3.15 ± 0.79	0.639	283	3.42 ± 0.93	0.983	669	3.26 ± 0.86	0.931
	1	409	3.12 ± 0.70		333	3.41 ± 0.85		742	3.26 ± 0.79	
	2	107	3.08 ± 0.77		90	3.43 ± 0.82		197	3.24 ± 0.81	
H2	0	490	3.15 ± 0.74	0.482	380	3.41 ± 0.84	0.917	870	3.26 ± 0.80	0.938
	1	346	3.10 ± 0.73		286	3.43 ± 0.93		632	3.25 ± 0.84	
	2	66	3.19 ± 0.86		40	3.41 ± 0.88		106	3.27 ± 0.87	
H3	0	670	3.10 ± 0.73	**0.015**	543	3.43 ± 0.87	0.716	1213	3.25 ± 0.82	0.114
	1	217	3.19 ± 0.77		148	3.37 ± 0.90		365	3.26 ± 0.83	
	2	15	3.61 ± 0.85		15	3.51 ± 0.75		30	3.56 ± 0.79	
H4	0	681	3.15 ± 0.77	0.402	530	3.44 ± 0.89	**0.043**	1211	3.28 ± 0.83	0.072
	1	207	3.08 ± 0.69		161	3.32 ± 0.80		368	3.18 ± 0.75	
	2	14	3.01 ± 0.74		15	3.86 ± 1.09		29	3.45 ± 1.02	
H5	0	727	3.12 ± 0.75	0.45	568	3.41 ± 0.86	0.855	1295	3.25 ± 0.81	0.21
	1	165	3.16 ± 0.72		135	3.44 ± 0.95		300	3.29 ± 0.84	

	2	10	3.40 ± 0.89		3	3.20 ± 1.08		13	3.35 ± 0.89
**HDL cholesterol (mmol/L)**									
H1	0	386	1.39 ± 0.30	0.22	283	1.16 ± 0.25	0.846	669	1.30 ± 0.30	0.59
	1	409	1.41 ± 0.31		333	1.17 ± 0.27		742	1.30 ± 0.30	
	2	107	1.35 ± 0.28		90	1.18 ± 0.28		197	1.28 ± 0.29	
H2	0	490	1.39 ± 0.31	0.31	380	1.16 ± 0.27	0.753	870	1.29 ± 0.31	0.116
	1	346	1.39 ± 0.28		286	1.17 ± 0.26		632	1.29 ± 0.29	
	2	66	1.45 ± 0.33		40	1.20 ± 0.31		106	1.36 ± 0.34	
H3	0	670	1.39 ± 0.30	0.674	543	1.17 ± 0.26	0.23	1213	1.29 ± 0.30	0.285
	1	217	1.41 ± 0.31		148	1.17 ± 0.28		365	1.31 ± 0.32	
	2	15	1.41 ± 0.29		15	1.05 ± 0.18		30	1.23 ± 0.30	
H4	0	681	1.40 ± 0.30	0.111	530	1.17 ± 0.27	0.731	1211	1.30 ± 0.31	0.135
	1	207	1.38 ± 0.30		161	1.18 ± 0.25		368	1.29 ± 0.30	
	2	14	1.26 ± 0.24		15	1.12 ± 0.27		29	1.19 ± 0.26	
H5	0	727	1.39 ± 0.30	0.571	568	1.17 ± 0.27	0.67	1295	1.30 ± 0.31	0.19
	1	165	1.40 ± 0.31		135	1.16 ± 0.25		300	1.29 ± 0.31	
	2	10	1.50 ± 0.39		3	1.29 ± 0.24		13	1.45 ± 0.36	
**Triglycerides (mmol/L)**										
H1	0	386	1.13 ± 0.54	0.59	283	1.39 ± 0.72	0.756	669	1.24 ± 0.64	0.515
	1	409	1.12 ± 0.49		333	1.43 ± 0.79		742	1.26 ± 0.66	
	2	107	1.18 ± 0.67		90	1.46 ± 1.17		197	1.31 ± 0.94	
H2	0	490	1.14 ± 0.56	0.581	380	1.41 ± 0.89	0.784	870	1.26 ± 0.73	0.456
	1	346	1.13 ± 0.51		286	1.44 ± 0.73		632	1.27 ± 0.63	
	2	66	1.07 ± 0.52		40	1.36 ± 0.88		106	1.18 ± 0.68	
H3	0	670	1.14 ± 0.55	0.594	543	1.41 ± 0.81	0.875	1213	1.26 ± 0.69	0.98
	1	217	1.13 ± 0.50		148	1.45 ± 0.85		365	1.26 ± 0.68	
	2	15	0.99 ± 0.32		15	1.47 ± 1.06		30	1.23 ± 0.81	
H4	0	681	1.12 ± 0.52	0.487	530	1.44 ± 0.88	0.507	1211	1.26 ± 0.72	0.938
	1	207	1.17 ± 0.59		161	1.35 ± 0.64		368	1.25 ± 0.62	
	2	14	1.04 ± 0.52		15	1.44 ± 0.59		29	1.25 ± 0.59	
H5	0	727	1.13 ± 0.54	0.702	568	1.42 ± 0.84	0.583	1295	1.26 ± 0.71	0.824
	1	165	1.16 ± 0.52		135	1.41 ± 0.74		300	1.27 ± 0.64	
	2	10	1.22 ± 0.45		3	0.93 ± 0.38		13	1.15 ± 0.44	
**Apolipoprotein B (g/L)**										

H1	0	386	1.00 ± 0.23	0.544	283	1.11 ± 0.26	0.982	669	1.05 ± 0.25	0.925
	1	409	0.98 ± 0.22		333	1.12 ± 0.25		742	1.04 ± 0.24	
	2	107	0.98 ± 0.24		90	1.12 ± 0.26		197	1.04 ± 0.26	
H2	0	490	0.99 ± 0.23	0.831	380	1.11 ± 0.25	0.874	870	1.04 ± 0.25	0.909
	1	346	0.98 ± 0.22		286	1.12 ± 0.25		632	1.05 ± 0.25	
	2	66	0.99 ± 0.25		40	1.11 ± 0.31		106	1.03 ± 0.28	
H3	0	670	0.98 ± 0.23	0.33	543	1.12 ± 0.26	0.781	1213	1.04 ± 0.25	0.36
	1	217	1.00 ± 0.24		148	1.11 ± 0.25		365	1.04 ± 0.25	
	2	15	1.06 ± 0.23		15	1.16 ± 0.20		30	1.11 ± 0.22	
H4	0	681	0.99 ± 0.23	0.687	530	1.12 ± 0.26	0.138	1211	1.05 ± 0.25	0.293
	1	207	0.98 ± 0.22		161	1.09 ± 0.24		368	1.03 ± 0.23	
	2	14	0.95 ± 0.22		15	1.20 ± 0.34		29	1.08 ± 0.31	
H5	0	727	0.98 ± 0.23	0.316	568	1.12 ± 0.25	0.751	1295	1.04 ± 0.25	0.567
	1	165	1.00 ± 0.23		135	1.12 ± 0.26		300	1.06 ± 0.25	
	2	10	1.06 ± 0.25		3	1.01 ± 0.30		13	1.05 ± 0.25	

Supplementary Table 4. The association of *USF1* polymorphisms and the lipid parameters in 2007 according to gender (The Cardiovascular Risk in Young Finns Study)

SNP no.	Genotype	N (women)	Mean ± SD	P	N (men)	Mean ± SD	P	N (all)	Mean ± SD	P
**Total cholesterol (mmol/L)**										
rs3737787	GG	376	4.96 ± 0.82	**0.039**	271	5.14 ± 0.91	0.248	647	5.03 ± 0.86	0.701
	GA	407	4.89 ± 0.81		324	5.13 ± 0.90		731	5.00 ± 0.86	
	AA	107	4.73 ± 0.77		89	5.31 ± 0.87		196	4.99 ± 0.86	
rs2516838	CC	462	4.94 ± 0.83	0.281	358	5.18 ± 0.90	0.576	820	5.05 ± 0.87	0.069
	CG	348	4.82 ± 0.75		279	5.12 ± 0.90		627	4.96 ± 0.83	
	GG	67	5.02 ± 0.86		41	5.26 ± 0.99		108	5.11 ± 0.92	
rs10908821	CC	663	4.89 ± 0.80	0.476	522	5.15 ± 0.89	0.956	1185	5.00 ± 0.85	0.67
	CG	207	4.92 ± 0.84		163	5.17 ± 0.95		370	5.03 ± 0.90	
	GG	15	4.67 ± 0.69		15	5.14 ± 0.91		30	4.90 ± 0.83	
rs2516839	TT	349	4.81 ± 0.80	**0.013**	282	5.20 ± 0.91	0.392	631	4.98 ± 0.87	0.254
	TC	419	4.93 ± 0.79		326	5.10 ± 0.89		745	5.01 ± 0.84	
	CC	123	5.03 ± 0.87		96	5.18 ± 0.90		219	5.10 ± 0.89	
rs1556259	AA	665	4.85 ± 0.80	**0.009**	536	5.16 ± 0.91	0.443	1201	4.99 ± 0.86	0.182
	AG	218	5.02 ± 0.82		150	5.07 ± 0.85		368	5.04 ± 0.83	
	GG	12	5.22 ± 0.97		13	5.31 ± 0.55		25	5.26 ± 0.77	
rs2774276	GG	517	4.87 ± 0.80	0.535	400	5.17 ± 0.89	0.889	917	5.00 ± 0.85	0.883
	GC	302	4.94 ± 0.81		243	5.14 ± 0.93		545	5.03 ± 0.87	
	CC	50	4.89 ± 0.87		40	5.16 ± 1.01		90	5.01 ± 0.94	
**LDL cholesterol (mmol/L)**										
rs3737787	GG	376	3.01 ± 0.69	**0.016**	271	3.27 ± 0.79	0.35	647	3.12 ± 0.75	0.432
	GA	407	2.91 ± 0.72		324	3.26 ± 0.80		731	3.06 ± 0.78	
	AA	107	2.80 ± 0.64		89	3.39 ± 0.75		196	3.07 ± 0.75	
rs2516838	CC	462	2.98 ± 0.72	0.12	358	3.30 ± 0.79	0.668	820	3.12 ± 0.77	0.17
	CG	348	2.88 ± 0.64		279	3.25 ± 0.80		627	3.05 ± 0.74	
	GG	67	3.01 ± 0.81		41	3.32 ± 0.84		108	3.13 ± 0.83	

rs10908821	CC	663	2.92 ± 0.70	0.744	522	3.27 ± 0.79	0.977	1185	3.08 ± 0.76	0.789
	CG	207	2.96 ± 0.69		163	3.28 ± 0.81		370	3.10 ± 0.76	
	GG	15	2.94 ± 0.60		15	3.31 ± 0.81		30	3.13 ± 0.73	
rs2516839	TT	349	2.85 ± 0.69	**0.002**	282	3.30 ± 0.80	0.619	631	3.05 ± 0.77	0.068
	TC	419	2.96 ± 0.69		326	3.24 ± 0.79		745	3.08 ± 0.75	
	CC	123	3.10 ± 0.73		96	3.31 ± 0.79		219	3.19 ± 0.76	
rs1556259	AA	665	2.89 ± 0.68	**0.003**	536	3.27 ± 0.79	0.276	1201	3.06 ± 0.76	**0.026**
	AG	218	3.05 ± 0.74		150	3.22 ± 0.78		368	3.12 ± 0.76	
	GG	12	3.30 ± 0.75		13	3.57 ± 0.56		25	3.44 ± 0.66	
rs2774276	GG	517	2.90 ± 0.71	0.223	400	3.28 ± 0.79	0.904	917	3.07 ± 0.77	0.631
	GC	302	2.99 ± 0.69		243	3.26 ± 0.80		545	3.11 ± 0.75	
	CC	50	2.96 ± 0.70		40	3.26 ± 0.86		90	3.09 ± 0.79	
**HDL cholesterol (mmol/L)**										
rs3737787	GG	376	1.44 ± 0.31	0.209	271	1.23 ± 0.29	0.322	647	1.35 ± 0.32	0.806
	GA	407	1.46 ± 0.31		324	1.20 ± 0.28		731	1.35 ± 0.32	
	AA	107	1.41 ± 0.35		89	1.24 ± 0.25		196	1.33 ± 0.32	
rs2516838	CC	462	1.44 ± 0.32	0.1	358	1.22 ± 0.27	0.306	820	1.35 ± 0.32	**0.016**
	CG	348	1.43 ± 0.28		279	1.20 ± 0.28		627	1.33 ± 0.30	
	GG	67	1.52 ± 0.35		41	1.26 ± 0.36		108	1.42 ± 0.38	
rs10908821	CC	663	1.46 ± 0.31	**0.041**	522	1.21 ± 0.28	0.409	1185	1.35 ± 0.32	0.255
	CG	207	1.43 ± 0.30		163	1.24 ± 0.28		370	1.35 ± 0.32	
	GG	15	1.27 ± 0.27		15	1.23 ± 0.23		30	1.25 ± 0.24	
rs2516839	TT	349	1.45 ± 0.32	0.524	282	1.21 ± 0.28	0.633	631	1.34 ± 0.33	0.904
	TC	419	1.45 ± 0.30		326	1.21 ± 0.28		745	1.35 ± 0.32	
	CC	123	1.42 ± 0.32		96	1.24 ± 0.27		219	1.34 ± 0.31	
rs1556259	AA	665	1.44 ± 0.31	0.429	536	1.23 ± 0.28	0.424	1201	1.34 ± 0.32	0.513
	AG	218	1.47 ± 0.32		150	1.19 ± 0.27		368	1.35 ± 0.32	
	GG	12	1.38 ± 0.30		13	1.19 ± 0.27		25	1.28 ± 0.29	
rs2774276	GG	517	1.46 ± 0.32	0.332	400	1.21 ± 0.28	0.15	917	1.35 ± 0.33	0.838
	GC	302	1.43 ± 0.29		243	1.23 ± 0.29		545	1.34 ± 0.30	
	CC	50	1.41 ± 0.34		40	1.30 ± 0.27		90	1.36 ± 0.31	

**Triglycerides (mmol/L)**										
rs3737787	GG	376	1.14 ± 0.54	0.905	271	1.43 ± 0.69	0.479	647	1.26 ± 0.62	0.418
	GA	407	1.14 ± 0.58		324	1.50 ± 0.74		731	1.30 ± 0.67	
	AA	107	1.17 ± 0.56		89	1.49 ± 0.69		196	1.31 ± 0.64	
rs2516838	CC	462	1.16 ± 0.59	0.565	358	1.45 ± 0.70	0.599	820	1.28 ± 0.66	0.811
	CG	348	1.13 ± 0.53		279	1.50 ± 0.72		627	1.29 ± 0.65	
	GG	67	1.09 ± 0.40		41	1.51 ± 0.76		108	1.25 ± 0.60	
rs10908821	CC	663	1.13 ± 0.53	0.339	522	1.48 ± 0.72	0.594	1185	1.29 ± 0.65	0.59
	CG	207	1.18 ± 0.65		163	1.44 ± 0.69		370	1.30 ± 0.68	
	GG	15	1.01 ± 0.37		15	1.33 ± 0.47		30	1.17 ± 0.45	
rs2516839	TT	349	1.14 ± 0.51	0.953	282	1.53 ± 0.74	0.127	631	1.31 ± 0.65	0.39
	TC	419	1.15 ± 0.58		326	1.44 ± 0.70		745	1.27 ± 0.65	
	CC	123	1.15 ± 0.58		96	1.39 ± 0.67		219	1.25 ± 0.63	
rs1556259	AA	665	1.15 ± 0.56	0.797	536	1.47 ± 0.71	0.402	1201	1.29 ± 0.65	0.589
	AG	218	1.12 ± 0.55		150	1.46 ± 0.73		368	1.26 ± 0.65	
	GG	12	1.20 ± 0.43		13	1.20 ± 0.50		25	1.20 ± 0.46	
rs2774276	GG	517	1.14 ± 0.52	0.931	400	1.50 ± 0.73	0.302	917	1.30 ± 0.65	0.715
	GC	302	1.15 ± 0.59		243	1.44 ± 0.70		545	1.28 ± 0.66	
	CC	50	1.16 ± 0.60		40	1.35 ± 0.63		90	1.24 ± 0.62	
**Apolipoprotein B (g/L)**										
rs3737787	GG	376	0.95 ± 0.23	0.087	271	1.09 ± 0.25	0.473	647	1.01 ± 0.25	0.846
	GA	407	0.93 ± 0.22		324	1.10 ± 0.25		731	1.00 ± 0.25	
	AA	107	0.91 ± 0.20		89	1.12 ± 0.23		196	1.01 ± 0.24	
rs2516838	CC	462	0.95 ± 0.23	0.287	358	1.09 ± 0.24	0.795	820	1.01 ± 0.24	0.652
	CG	348	0.92 ± 0.21		279	1.10 ± 0.25		627	1.00 ± 0.25	
	GG	67	0.95 ± 0.24		41	1.12 ± 0.28		108	1.01 ± 0.27	
rs10908821	CC	663	0.93 ± 0.22	0.521	522	1.10 ± 0.24	0.894	1185	1.00 ± 0.24	0.881
	CG	207	0.95 ± 0.24		163	1.09 ± 0.26		370	1.01 ± 0.25	
	GG	15	0.93 ± 0.18		15	1.08 ± 0.27		30	1.01 ± 0.24	
rs2516839	TT	349	0.91 ± 0.22	**0.015**	282	1.11 ± 0.25	0.222	631	1.00 ± 0.25	0.46
	TC	419	0.94 ± 0.22		326	1.08 ± 0.24		745	1.00 ± 0.24	

	CC	123	0.98 ± 0.24		96	1.08 ± 0.25		219	1.02 ± 0.25	
rs1556259	AA	665	0.93 ± 0.22	**0.049**	536	1.10 ± 0.25	0.638	1201	1.00 ± 0.25	0.388
	AG	218	0.96 ± 0.23		150	1.08 ± 0.23		368	1.00 ± 0.24	
	GG	12	1.05 ± 0.24		13	1.09 ± 0.20		25	1.07 ± 0.22	
rs2774276	GG	517	0.93 ± 0.22	0.283	400	1.10 ± 0.25	0.581	917	1.00 ± 0.25	0.872
	GC	302	0.95 ± 0.23		243	1.08 ± 0.25		545	1.01 ± 0.25	
	CC	50	0.95 ± 0.23		40	1.08 ± 0.28		90	1.00 ± 0.26	

Supplementary Table 5. The association of *USF1* haplotypes and lipid parameters in 2007 according to gender (The Cardiovascular Risk in Young Finns Study)

Haplotype no.	copy	N (women)	Mean ± SD	P	N (men)	Mean ± SD	P	N (all)	Mean ± SD	P
**Total cholesterol (mmol/L)**										
H1	0	386	4.96 ± 0.81	**0.025**	283	5.15 ± 0.90	0.592	669	5.04 ± 0.86	0.419
	1	409	4.89 ± 0.81		333	5.13 ± 0.91		742	5.00 ± 0.86	
	2	107	4.72 ± 0.77		90	5.24 ± 0.87		197	4.96 ± 0.85	
H2	0	490	4.94 ± 0.84	0.289	380	5.16 ± 0.90	0.532	870	5.04 ± 0.87	0.076
	1	346	4.82 ± 0.74		286	5.12 ± 0.89		632	4.95 ± 0.82	
	2	66	5.00 ± 0.87		40	5.28 ± 1.00		106	5.11 ± 0.93	
H3	0	670	4.85 ± 0.80	**0.006**	543	5.17 ± 0.92	0.336	1213	4.99 ± 0.87	0.129
	1	217	5.02 ± 0.82		148	5.07 ± 0.85		365	5.04 ± 0.83	
	2	15	5.23 ± 0.93		15	5.34 ± 0.63		30	5.29 ± 0.78	
H4	0	681	4.90 ± 0.80	0.599	530	5.15 ± 0.88	0.993	1211	5.01 ± 0.85	0.819
	1	207	4.92 ± 0.84		161	5.16 ± 0.95		368	5.03 ± 0.90	
	2	14	4.70 ± 0.70		15	5.14 ± 0.91		29	4.93 ± 0.83	
H5	0	727	4.88 ± 0.81	0.173	568	5.16 ± 0.89	0.757	1295	5.00 ± 0.86	0.442
	1	165	4.95 ± 0.78		135	5.10 ± 0.92		300	5.02 ± 0.85	
	2	10	5.31 ± 0.86		3	5.30 ± 1.00		13	5.31 ± 0.84	
**LDL cholesterol (mmol/L)**										
H1	0	386	3.01 ± 0.69	**0.01**	283	3.28 ± 0.79	0.651	669	3.12 ± 0.74	0.256
	1	409	2.91 ± 0.72		333	3.25 ± 0.81		742	3.06 ± 0.78	
	2	107	2.79 ± 0.64		90	3.34 ± 0.76		197	3.04 ± 0.74	
H2	0	490	2.97 ± 0.72	0.106	380	3.29 ± 0.80	0.611	870	3.11 ± 0.77	0.163
	1	346	2.88 ± 0.63		286	3.24 ± 0.79		632	3.04 ± 0.73	
	2	66	3.00 ± 0.82		40	3.33 ± 0.84		106	3.13 ± 0.84	
H3	0	670	2.89 ± 0.68	**0.003**	543	3.28 ± 0.80	0.16	1213	3.07 ± 0.76	**0.022**
	1	217	3.05 ± 0.74		148	3.21 ± 0.78		365	3.12 ± 0.76	
	2	15	3.26 ± 0.75		15	3.60 ± 0.64		30	3.43 ± 0.71	
H4	0	681	2.93 ± 0.70	0.748	530	3.27 ± 0.79	0.979	1211	3.08 ± 0.76	0.822
	1	207	2.97 ± 0.70		161	3.27 ± 0.81		368	3.10 ± 0.76	
	2	14	2.96 ± 0.61		15	3.31 ± 0.81		29	3.14 ± 0.73	
H5	0	727	2.93 ± 0.70	0.235	568	3.28 ± 0.79	0.785	1295	3.08 ± 0.76	0.569
	1	165	2.97 ± 0.68		135	3.23 ± 0.80		300	3.09 ± 0.75	

	2	10	3.28 ± 0.55		3	3.39 ± 0.89		13	3.31 ± 0.60	
**HDL cholesterol (mmol/L)**										
H1	0	386	1.44 ± 0.31	0.237	283	1.23 ± 0.29	0.385	669	1.35 ± 0.32	0.59
	1	409	1.46 ± 0.30		333	1.20 ± 0.28		742	1.35 ± 0.32	
	2	107	1.41 ± 0.35		90	1.23 ± 0.25		197	1.33 ± 0.32	
H2	0	490	1.45 ± 0.32	0.144	380	1.22 ± 0.27	0.395	870	1.35 ± 0.32	**0.023**
	1	346	1.43 ± 0.29		286	1.20 ± 0.28		632	1.33 ± 0.30	
	2	66	1.51 ± 0.35		40	1.26 ± 0.36		106	1.42 ± 0.37	
H3	0	670	1.44 ± 0.31	0.562	543	1.23 ± 0.28	0.409	1213	1.34 ± 0.32	0.758
	1	217	1.47 ± 0.32		148	1.19 ± 0.27		365	1.35 ± 0.33	
	2	15	1.44 ± 0.30		15	1.19 ± 0.25		30	1.32 ± 0.30	
H4	0	681	1.46 ± 0.32	0.057	530	1.21 ± 0.28	0.474	1211	1.35 ± 0.32	0.326
	1	207	1.42 ± 0.30		161	1.24 ± 0.28		368	1.34 ± 0.30	
	2	14	1.30 ± 0.26		15	1.23 ± 0.23		29	1.26 ± 0.24	
H5	0	727	1.44 ± 0.31	0.819	568	1.21 ± 0.27	0.44	1295	1.34 ± 0.32	0.447
	1	165	1.46 ± 0.29		135	1.24 ± 0.31		300	1.36 ± 0.32	
	2	10	1.45 ± 0.36		3	1.34 ± 0.25		13	1.42 ± 0.33	
**Triglycerides (mmol/L)**										
H1	0	386	1.14 ± 0.53	0.895	283	1.42 ± 0.68	0.428	669	1.26 ± 0.61	0.364
	1	409	1.14 ± 0.58		333	1.50 ± 0.74		742	1.30 ± 0.68	
	2	107	1.16 ± 0.56		90	1.49 ± 0.69		197	1.31 ± 0.64	
H2	0	490	1.16 ± 0.59	0.646	380	1.44 ± 0.69	0.531	870	1.28 ± 0.65	0.726
	1	346	1.13 ± 0.53		286	1.50 ± 0.73		632	1.30 ± 0.65	
	2	66	1.09 ± 0.39		40	1.51 ± 0.76		106	1.25 ± 0.59	
H3	0	670	1.15 ± 0.56	0.867	543	1.47 ± 0.70	0.34	1213	1.29 ± 0.65	0.555
	1	217	1.13 ± 0.55		148	1.48 ± 0.75		365	1.27 ± 0.66	
	2	15	1.16 ± 0.40		15	1.20 ± 0.47		30	1.18 ± 0.43	
H4	0	681	1.13 ± 0.53	0.262	530	1.48 ± 0.72	0.626	1211	1.28 ± 0.64	0.548
	1	207	1.19 ± 0.65		161	1.44 ± 0.69		368	1.30 ± 0.68	
	2	14	0.98 ± 0.36		15	1.33 ± 0.47		29	1.16 ± 0.45	
H5	0	727	1.14 ± 0.55	0.638	568	1.48 ± 0.71	0.404	1295	1.29 ± 0.65	0.838
	1	165	1.16 ± 0.58		135	1.40 ± 0.70		300	1.27 ± 0.65	
	2	10	1.30 ± 0.74		3	1.26 ± 0.64		13	1.29 ± 0.69	
**Apolipoprotein B (g/L)**										

H1	0	386	0.95 ± 0.23	0.069	283	1.09 ± 0.25	0.701	669	1.01 ± 0.25	0.78
	1	409	0.93 ± 0.22		333	1.09 ± 0.25		742	1.00 ± 0.25	
	2	107	0.91 ± 0.20		90	1.11 ± 0.23		197	1.00 ± 0.24	
H2	0	490	0.95 ± 0.23	0.311	380	1.09 ± 0.24	0.661	870	1.01 ± 0.24	0.741
	1	346	0.92 ± 0.21		286	1.09 ± 0.25		632	1.00 ± 0.25	
	2	66	0.94 ± 0.24		40	1.13 ± 0.28		106	1.01 ± 0.27	
H3	0	670	0.93 ± 0.22	0.055	543	1.10 ± 0.25	0.621	1213	1.00 ± 0.25	0.421
	1	217	0.96 ± 0.23		148	1.08 ± 0.23		365	1.00 ± 0.24	
	2	15	1.03 ± 0.23		15	1.09 ± 0.19		30	1.06 ± 0.21	
H4	0	681	0.93 ± 0.22	0.465	530	1.10 ± 0.24	0.872	1211	1.00 ± 0.24	0.873
	1	207	0.95 ± 0.24		161	1.09 ± 0.26		368	1.01 ± 0.25	
	2	14	0.93 ± 0.19		15	1.08 ± 0.27		29	1.01 ± 0.24	
H5	0	727	0.93 ± 0.22	0.248	568	1.10 ± 0.25	0.612	1295	1.00 ± 0.25	0.747
	1	165	0.94 ± 0.22		135	1.07 ± 0.24		300	1.00 ± 0.24	
	2	10	1.04 ± 0.24		3	1.10 ± 0.35		13	1.06 ± 0.25	

Supplementary Table 6. The association of *USF1* rs2516839, rs1556259, haplotype 3 and serum lipoprotein subclass cholesterol fractions (total cholesterol, cholesterol ester, and free cholesterol when available) and total lipid concentrations in 2007 measured by ^{1}H-NMR spectroscopy in women (The Cardiovascular Risk in Young Finns Study)

	rs2516839				rs1556259				Haplotype 3			
	TT	TC	CC	P	AA	AG	GG	P	0	1	2	P
	349	419	123		665	218	12		670	217	15	
Total cholesterol (mmol/L)												
L_VLDL	0.03±0.03	0.04±0.03	0.04±0.04	0.332	0.04±0.04	0.04±0.04	0.04±0.02	0.95	0.04±0.04	0.04±0.04	0.03±0.02	0.897
M_VLDL	0.13±0.06	0.13±0.06	0.14±0.07	0.177	0.13±0.06	0.13±0.07	0.16±0.06	0.484	0.13±0.06	0.13±0.07	0.15±0.06	0.658
S_VLDL	0.21±0.07	0.21±0.08	0.23±0.08	**0.008**	0.21±0.08	0.22±0.08	0.27±0.10	**0.014**	0.21±0.08	0.22±0.08	0.26±0.10	**0.013**
L_LDL	0.96±0.25	0.99±0.25	1.05±0.26	**0.001**	0.97±0.25	1.03±0.26	1.18±0.32	**<0.001**	0.97±0.25	1.03±0.26	1.19±0.35	**<0.001**
M_LDL	0.54±0.16	0.57±0.16	0.61±0.17	**0.001**	0.55±0.16	0.59±0.17	0.68±0.19	**0.001**	0.55±0.16	0.59±0.17	0.69±0.22	**<0.001**
S_LDL	0.33±0.10	0.34±0.10	0.37±0.11	**0.001**	0.33±0.10	0.35±0.11	0.40±0.11	**0.002**	0.33±0.10	0.35±0.11	0.40±0.13	**0.001**
XL_HDL	0.20±0.11	0.21±0.11	0.18±0.10	**0.037**	0.20±0.11	0.21±0.11	0.15±0.08	0.195	0.20±0.11	0.21±0.11	0.16±0.08	0.251
L_HDL	0.48±0.23	0.50±0.22	0.44±0.21	**0.036**	0.48±0.22	0.50±0.23	0.43±0.23	0.477	0.48±0.22	0.49±0.23	0.47±0.22	0.674
M_HDL	0.55±0.13	0.57±0.15	0.56±0.14	0.345	0.56±0.14	0.57±0.15	0.57±0.19	0.545	0.56±0.14	0.57±0.15	0.59±0.19	0.343
Free cholesterol (mmol/L)												
L_VLDL	0.02±0.02	0.02±0.02	0.02±0.02	0.268	0.02±0.02	0.02±0.02	0.02±0.01	0.975	0.02±0.02	0.02±0.02	0.02±0.01	0.956
M_VLDL	0.05±0.03	0.05±0.03	0.06±0.03	0.224	0.05±0.03	0.05±0.03	0.06±0.03	0.671	0.05±0.03	0.05±0.03	0.06±0.02	0.832
S_VLDL	0.08±0.03	0.08±0.03	0.09±0.03	**0.022**	0.08±0.03	0.08±0.03	0.10±0.04	0.052	0.08±0.03	0.08±0.03	0.10±0.03	0.064
IDL	0.22±0.06	0.23±0.05	0.24±0.06	**0.008**	0.23±0.05	0.24±0.05	0.27±0.07	**0.001**	0.23±0.05	0.24±0.05	0.27±0.07	**<0.001**
L_LDL	0.27±0.06	0.28±0.06	0.29±0.07	**0.003**	0.27±0.06	0.29±0.06	0.32±0.08	**0.001**	0.27±0.06	0.29±0.06	0.33±0.08	**<0.001**
XL_HDL	0.06±0.03	0.06±0.03	0.05±0.03	**0.038**	0.06±0.03	0.06±0.03	0.05±0.03	0.303	0.06±0.03	0.06±0.03	0.05±0.03	0.396
L_HDL	0.11±0.05	0.11±0.05	0.10±0.05	**0.041**	0.10±0.05	0.11±0.05	0.10±0.06	0.601	0.10±0.05	0.11±0.05	0.11±0.05	0.794
M_HDL	0.11±0.03	0.11±0.03	0.11±0.03	0.291	0.11±0.03	0.11±0.03	0.11±0.04	0.43	0.11±0.03	0.11±0.03	0.12±0.04	0.179
Cholesterol ester (mmol/L)												
L_VLDL	0.02±0.02	0.02±0.02	0.02±0.02	0.391	0.02±0.02	0.02±0.02	0.02±0.01	0.915	0.02±0.02	0.02±0.02	0.02±0.01	0.84
M_VLDL	0.08±0.03	0.08±0.03	0.08±0.04	0.161	0.08±0.03	0.08±0.03	0.09±0.03	0.307	0.08±0.03	0.08±0.03	0.09±0.03	0.444
L_LDL	0.69±0.19	0.71±0.19	0.76±0.20	**0.001**	0.70±0.19	0.74±0.20	0.86±0.24	**<0.001**	0.70±0.19	0.74±0.20	0.86±0.26	**<0.001**
M_LDL	0.39±0.13	0.41±0.13	0.45±0.13	**0.001**	0.40±0.13	0.43±0.13	0.50±0.15	**0.001**	0.40±0.13	0.43±0.13	0.51±0.17	**<0.001**
XL_HDL	0.15±0.08	0.15±0.08	0.13±0.07	**0.035**	0.14±0.08	0.15±0.07	0.11±0.05	0.195	0.14±0.08	0.15±0.07	0.12±0.05	0.233
L_HDL	0.38±0.18	0.39±0.17	0.34±0.16	**0.035**	0.37±0.17	0.39±0.18	0.34±0.18	0.441	0.37±0.17	0.39±0.18	0.36±0.17	0.628
M_HDL	0.45±0.11	0.46±0.12	0.46±0.11	0.361	0.45±0.11	0.46±0.12	0.46±0.15	0.558	0.45±0.11	0.46±0.12	0.48±0.15	0.38
Total lipids (mmol/L)												

XXL_VLDL	0.01±0.02	0.01±0.02	0.01±0.02	0.505	0.01±0.02	0.01±0.02	0.01±0.01	0.874	0.01±0.02	0.01±0.02	0.01±0.01	0.689
XL_VLDL	0.03±0.04	0.04±0.04	0.04±0.05	0.242	0.04±0.05	0.04±0.05	0.03±0.03	0.961	0.04±0.05	0.04±0.05	0.03±0.03	0.795
L_VLDL	0.17±0.16	0.17±0.16	0.19±0.19	0.335	0.17±0.17	0.17±0.17	0.17±0.10	0.891	0.17±0.17	0.17±0.17	0.17±0.10	0.836
M_VLDL	0.43±0.24	0.44±0.25	0.48±0.28	0.257	0.44±0.25	0.44±0.26	0.50±0.18	0.706	0.44±0.25	0.44±0.26	0.50±0.18	0.842
S_VLDL	0.52±0.19	0.54±0.20	0.58±0.21	**0.041**	0.53±0.20	0.54±0.21	0.65±0.22	0.127	0.53±0.20	0.54±0.21	0.65±0.22	0.163
XS_VLDL	0.51±0.14	0.53±0.15	0.54±0.14	0.109	0.52±0.14	0.53±0.14	0.62±0.17	**0.022**	0.52±0.14	0.53±0.14	0.62±0.17	**0.032**
IDL	1.20±0.28	1.24±0.28	1.28±0.28	**0.011**	1.21±0.28	1.26±0.28	1.44±0.35	**0.001**	1.21±0.28	1.26±0.28	1.44±0.35	**0.001**
L_LDL	1.42±0.36	1.47±0.35	1.55±0.37	**0.002**	1.44±0.35	1.51±0.36	1.73±0.44	**0.001**	1.44±0.35	1.51±0.36	1.73±0.44	**<0.001**
M_LDL	0.81±0.22	0.84±0.22	0.89±0.23	**0.001**	0.82±0.22	0.87±0.23	0.99±0.26	**0.002**	0.82±0.22	0.87±0.23	0.99±0.26	**<0.001**
S_LDL	0.51±0.14	0.53±0.14	0.56±0.16	**0.001**	0.52±0.14	0.55±0.15	0.61±0.15	**0.004**	0.52±0.14	0.55±0.15	0.61±0.15	**0.001**
XL_HDL	0.48±0.25	0.48±0.24	0.41±0.21	**0.022**	0.47±0.25	0.48±0.24	0.38±0.19	0.369	0.47±0.25	0.48±0.24	0.38±0.19	0.571
L_HDL	1.02±0.42	1.05±0.40	0.94±0.40	**0.049**	1.02±0.40	1.04±0.43	0.97±0.45	0.64	1.02±0.40	1.04±0.43	0.97±0.45	0.737
M_HDL	1.09±0.24	1.12±0.26	1.11±0.25	0.41	1.10±0.25	1.12±0.26	1.14±0.33	0.658	1.10±0.25	1.12±0.26	1.14±0.33	0.37
S_HDL	1.20±0.15	1.22±0.16	1.25±0.15	**0.013**	1.21±0.16	1.22±0.15	1.28±0.19	0.187	1.21±0.16	1.22±0.15	1.28±0.19	**0.033**

Supplementary Table 7. The association of *USF1* rs2516839, rs1556259, haplotype 3 and serum lipoprotein subclass cholesterol fractions (total cholesterol, cholesterol ester, and free cholesterol when available) and total lipid concentrations in 2001 measured by ^{1}H-NMR spectroscopy in women (The Cardiovascular Risk in Young Finns Study)

	rs2516839				rs1556259				Haplotype 3			
	TT	TC	CC	P	AA	AG	GG	P	0	1	2	P
	349	419	123		665	218	12		670	217	15	
Total cholesterol (mmol/L)												
L_VLDL	0.04±0.04	0.04±0.04	0.04±0.04	0.91	0.04±0.04	0.04±0.03	0.02±0.02	0.338	0.04±0.04	0.04±0.03	0.03±0.02	0.498
M_VLDL	0.13±0.07	0.13±0.07	0.14±0.07	0.647	0.13±0.07	0.13±0.07	0.12±0.05	0.856	0.13±0.07	0.13±0.07	0.13±0.05	0.922
S_VLDL	0.21±0.08	0.22±0.09	0.22±0.08	0.164	0.21±0.08	0.22±0.09	0.22±0.08	0.608	0.21±0.08	0.22±0.09	0.23±0.07	0.402
L_LDL	0.96±0.26	0.99±0.27	1.00±0.27	0.171	0.96±0.26	1.01±0.28	1.14±0.29	**0.009**	0.96±0.26	1.01±0.28	1.11±0.29	**0.008**
M_LDL	0.56±0.16	0.57±0.17	0.58±0.17	0.267	0.56±0.16	0.59±0.18	0.66±0.17	**0.013**	0.56±0.16	0.59±0.18	0.65±0.17	**0.012**
S_LDL	0.33±0.10	0.34±0.10	0.35±0.10	0.364	0.34±0.10	0.35±0.11	0.39±0.10	**0.031**	0.34±0.10	0.35±0.11	0.39±0.10	**0.026**
XL_HDL	0.20±0.11	0.20±0.11	0.19±0.11	0.628	0.19±0.11	0.21±0.11	0.20±0.09	0.462	0.19±0.11	0.21±0.11	0.20±0.08	0.458
L_HDL	0.46±0.21	0.47±0.20	0.44±0.22	0.342	0.46±0.21	0.48±0.21	0.42±0.18	0.211	0.46±0.21	0.48±0.21	0.42±0.17	0.228
M_HDL	0.54±0.12	0.56±0.15	0.55±0.14	0.527	0.55±0.13	0.56±0.16	0.52±0.11	0.425	0.55±0.13	0.56±0.16	0.53±0.12	0.433
Free cholesterol (mmol/L)												
L_VLDL	0.02±0.02	0.02±0.02	0.02±0.02	0.886	0.02±0.02	0.02±0.02	0.01±0.01	0.312	0.02±0.02	0.02±0.02	0.01±0.01	0.428
M_VLDL	0.05±0.03	0.06±0.03	0.06±0.03	0.692	0.06±0.03	0.06±0.03	0.05±0.02	0.701	0.06±0.03	0.06±0.03	0.05±0.02	0.832
S_VLDL	0.08±0.03	0.08±0.03	0.08±0.03	0.184	0.08±0.03	0.08±0.03	0.08±0.03	0.765	0.08±0.03	0.08±0.03	0.09±0.03	0.589
IDL	0.21±0.05	0.22±0.06	0.22±0.06	0.102	0.22±0.06	0.23±0.06	0.26±0.06	**0.004**	0.22±0.06	0.23±0.06	0.25±0.06	**0.005**
L_LDL	0.27±0.06	0.27±0.07	0.28±0.07	0.189	0.27±0.06	0.28±0.07	0.32±0.07	**0.004**	0.27±0.06	0.28±0.07	0.31±0.07	**0.004**
XL_HDL	0.06±0.03	0.06±0.03	0.05±0.03	0.585	0.06±0.03	0.06±0.03	0.05±0.03	0.294	0.06±0.03	0.06±0.03	0.06±0.02	0.323
L_HDL	0.10±0.05	0.10±0.05	0.09±0.05	0.398	0.10±0.05	0.10±0.05	0.09±0.04	0.208	0.10±0.05	0.10±0.05	0.09±0.04	0.227
M_HDL	0.10±0.02	0.11±0.03	0.11±0.03	0.409	0.10±0.03	0.11±0.03	0.10±0.02	0.414	0.10±0.03	0.11±0.03	0.10±0.02	0.406
Cholesterol ester (mmol/L)												
L_VLDL	0.02±0.02	0.02±0.02	0.02±0.02	0.921	0.02±0.02	0.02±0.02	0.01±0.01	0.369	0.02±0.02	0.02±0.02	0.01±0.01	0.568
M_VLDL	0.08±0.04	0.08±0.04	0.08±0.03	0.619	0.08±0.04	0.08±0.04	0.07±0.03	0.901	0.08±0.04	0.08±0.04	0.08±0.03	0.868
L_LDL	0.69±0.19	0.71±0.20	0.72±0.20	0.169	0.69±0.19	0.73±0.21	0.82±0.22	**0.013**	0.69±0.19	0.73±0.21	0.79±0.22	**0.011**
M_LDL	0.41±0.13	0.42±0.13	0.42±0.13	0.291	0.41±0.13	0.43±0.14	0.49±0.14	**0.016**	0.41±0.13	0.43±0.14	0.47±0.14	**0.014**
XL_HDL	0.14±0.08	0.14±0.08	0.13±0.08	0.558	0.14±0.08	0.15±0.08	0.14±0.06	0.439	0.14±0.08	0.15±0.08	0.14±0.06	0.431
L_HDL	0.36±0.16	0.37±0.16	0.34±0.18	0.323	0.36±0.16	0.38±0.16	0.33±0.14	0.211	0.36±0.16	0.38±0.16	0.33±0.13	0.228
M_HDL	0.44±0.09	0.45±0.12	0.45±0.11	0.554	0.44±0.10	0.45±0.13	0.42±0.09	0.412	0.44±0.10	0.45±0.13	0.42±0.09	0.422
Total lipids (mmol/L)												

XXL_VLDL	0.01±0.02	0.01±0.02	0.01±0.02	0.943	0.01±0.02	0.01±0.02	0.01±0.01	0.39	0.01±0.02	0.01±0.02	0.01±0.01	0.416
XL_VLDL	0.04±0.05	0.04±0.05	0.04±0.05	0.94	0.04±0.05	0.03±0.04	0.02±0.02	0.273	0.04±0.05	0.04±0.04	0.02±0.02	0.367
L_VLDL	0.19±0.19	0.18±0.16	0.20±0.18	0.779	0.19±0.18	0.18±0.16	0.12±0.08	0.384	0.19±0.18	0.18±0.16	0.14±0.18	0.499
M_VLDL	0.46±0.28	0.46±0.24	0.48±0.26	0.716	0.46±0.26	0.46±0.25	0.41±0.16	0.778	0.46±0.26	0.46±0.25	0.43±0.16	0.884
S_VLDL	0.53±0.22	0.55±0.21	0.56±0.20	0.352	0.55±0.21	0.55±0.22	0.56±0.20	0.868	0.54±0.21	0.56±0.22	0.57±0.19	0.729
XS_VLDL	0.48±0.14	0.50±0.15	0.50±0.13	0.08	0.49±0.14	0.50±0.15	0.56±0.17	0.09	0.49±0.14	0.50±0.15	0.55±0.16	0.078
IDL	1.15±0.28	1.19±0.31	1.19±0.29	0.111	1.16±0.28	1.21±0.31	1.37±0.35	**0.008**	1.16±0.28	1.21±0.31	1.33±0.34	**0.009**
L_LDL	1.41±0.35	1.45±0.38	1.47±0.37	0.172	1.42±0.36	1.48±0.39	1.67±0.41	**0.008**	1.42±0.36	1.49±0.39	1.63±0.40	**0.008**
M_LDL	0.82±0.22	0.84±0.23	0.86±0.23	0.245	0.83±0.22	0.86±0.24	0.97±0.24	**0.014**	0.83±0.22	0.87±0.24	0.95±0.24	**0.013**
S_LDL	0.52±0.15	0.53±0.15	0.54±0.15	0.316	0.52±0.15	0.54±0.15	0.60±0.15	**0.04**	0.52±0.15	0.54±0.15	0.59±0.15	**0.036**
XL_HDL	0.45±0.23	0.46±0.23	0.43±0.24	0.558	0.45±0.23	0.47±0.24	0.44±0.20	0.369	0.45±0.23	0.47±0.24	0.44±0.18	0.412
L_HDL	0.97±0.39	0.98±0.39	0.93±0.41	0.417	0.96±0.39	1.01±0.41	0.92±0.32	0.64	0.96±0.39	1.01±0.41	0.91±0.31	0.257
M_HDL	1.07±0.22	1.09±0.27	1.10±0.26	0.524	1.08±0.23	1.10±0.29	1.05±0.18	0.658	1.08±0.23	1.10±0.29	1.06±0.21	0.54
S_HDL	1.23±0.14	1.24±0.17	1.26±0.17	0.19	1.23±0.15	1.25±0.18	1.27±0.13	0.187	1.23±0.15	1.25±0.18	1.27±0.15	0.313

Supplementary Methods:

Vascular samples—Tampere Vascular Study

The atherosclerotic vascular samples used in this study (N=68) were obtained from patients subjected to carotid (N=29) or femoral (N=24) endarterectomy due to symptomatic stenosis or abdominal aortic (N=15) bypass procedure (i.e., aortobifemoral bypass) due to aortoiliac atherosclerosis and the left internal thoracic arteries (LITA, control samples) (N=23) samples were obtained during coronary artery bypass surgery, as part of ongoing Tampere Vascular Study (TVS) {Levula, 2009 #1218; Niiinisalo, 2010 #1217; Oksala, 2009 #1185; Oksala, 2010 #1209}. All open vascular surgical procedures were performed at the Division of Vascular Surgery and Heart Center, Tampere University Hospital. The study was approved by the Ethics Committee of Tampere University Hospital, and the study subjects gave their informed consent. The protocols were carried out in accordance with the approved guidelines. The vascular samples were classified according to recommendation of American Heart Association (AHA) {Stary, 1995 #410}. All the internal mammary arteries that were used as controls were verified to be microscopically healthy.

RNA isolation and genome-wide expression analysis

Endarterectomy samples constituting the intima and inner media from carotid, femoral and aortic regions were obtained. The harvested samples were 5-10 mm x 5-10 mm in size. These fresh tissue samples were immediately soaked in RNALater solution (Ambion Inc., Austin, TX, USA), and RNA was extracted with the Trizol reagent (Invitrogen, Carlsbad, CA, USA) and the RNEasy® Kit with RNase-Free DNase Set (Qiagen, Inc, Hilden, Germany). RNA and cRNA quality control was carried out using the Bioanalyzer (Agilent Santa, Clara CA, USA) and quantification was done using Ribogreen (Invitrogen). 300-500ng of RNA was reverse transcribed into cRNA and

biotin-UTP labeled using the Illumina TotalPrep RNA Amplification Kit (Ambion). 1,500 ng of cRNA was hybridized to the Illumina HumanHT-12 v3 Expression BeadChip. The chips were incubated over night, and washing steps and detection were carried out according to manufacturer's instructions.

BeadChips were scanned with the Illumina iScan system. After background subtraction raw intensity data was exported using Illumina GenomeStudio software. Further data processing was conducted using R language and appropriate Bioconductor modules. Robust multiarray averaging (RMA) was used to correct negative intensity values after background subtraction. RMA was the method of choice because it produces differentially expressed genes with highest correlation with qRT-PCR {Schmid, 2010 #1193}. Between arrays normalization was done using robust spline normalization (RSN) which combines the most desired properties of loess and quantile normalizations {Barnes, 2005 #1194}. Quality control was performed using sample clustering and multi dimensional scaling. Outliers were removed due to low expression profiles. Statistical significance was tested using Kruskal–Wallis test. Findings were considered statistically significant at $P < 0.05$.

DNA extraction and genotyping of the *USF1* polymorphisms

Briefly, 200ng of DNA was amplified with Multi-Sample Amplification Master Mix (Illumina), enzymatically fragmented, precipitated, resuspended and transferred to the chip. The samples were hybridized to the Beadchip and primers incorporated during hybridization were extended with labeled nucleotides. The hybridized DNA was removed and extended primers were stained. Dried Beadchip was scanned with iScan system. Genotype calling was performed using the standard procedure implemented in GenomeStudio, with the default parameters suggested by the platform manufacturer (Illumina). Genotypes of six SNPs (rs3737787, rs2516838, rs10908821, rs2516839,

rs1556259, rs2774276) were imputed with MACH version 1.0 {Li, 2009 #1195} from genotyped autosomal SNPs, that passed quality control and were present on HapMap.
